# Targeting MICA/B with cytotoxic therapeutic antibodies leads to tumor control [version 2; peer review: 2 approved]

**DOI:** 10.12688/openreseurope.13314.1

**Published:** 2021-10-27

**Authors:** Mathieu Bléry, Manel Mrabet-Kraiem, Ariane Morel, Florence Lhospice, Delphine Bregeon, Cécile Bonnafous, Laurent Gauthier, Benjamin Rossi, Romain Remark, Stéphanie Cornen, Nadia Anceriz, Nicolas Viaud, Sylvia Trichard, Sabrina Carpentier, Alix Joulin-Giet, Gwendoline Grondin, Veronika Liptakova, Younghoon Kim, Laurent Daniel, Aurélie Haffner, Nicolas Macagno, Laurent Pouyet, Ivan Perrot, Carine Paturel, Yannis Morel, Alexander Steinle, François Romagné, Emilie Narni-Mancinelli, Eric Vivier

**Affiliations:** 1 Innate Pharma, Marseille, France; 2 Institute for Molecular Medicine, Goethe-University Frankfurt am Main, Frankfurt am Main, Germany; 3 Assistance Publique des Hôpitaux de Marseille, Hôpital de la Timone, Marseille, France; 4 MI-mAbs, Aix Marseille University, Marseille, France; 5 Frankfurt Cancer Institute, Frankfurt am Main, Germany; 6 Aix Marseille University, CNRS, INSERM, CIML, Marseille, France

**Keywords:** MICA, ADC, cancer immunotherapy

## Abstract

**Background:**

MICA and MICB are tightly regulated stress-induced proteins that trigger the immune system by binding to the activating receptor NKG2D on cytotoxic lymphocytes. MICA and MICB are highly polymorphic molecules with prevalent expression on several types of solid tumors and limited expression in normal/healthy tissues, making them attractive targets for therapeutic intervention.

**Methods:**

We have generated a series of anti-MICA and MICB cross-reactive antibodies with the unique feature of binding to the most prevalent isoforms of both these molecules.

**Results:**

The anti-MICA and MICB antibody MICAB1, a human IgG1 Fc-engineered monoclonal antibody (mAb), displayed potent antibody-dependent cellular cytotoxicity (ADCC) and antibody-dependent cellular phagocytosis (ADCP) of MICA/B-expressing tumor cells *in vitro*. However, it showed insufficient efficiency against solid tumors *in vivo*, which prompted the development of antibody-drug conjugates (ADC). Indeed, optimal tumor control was achieved with MICAB1-ADC format in several solid tumor models, including patient-derived xenografts (PDX) and carcinogen-induced tumors in immunocompetent MICAgen transgenic mice.

**Conclusions:**

These data indicate that MICA and MICB are promising targets for cytotoxic immunotherapy.

## Introduction

Cancer is one of the most feared diseases of the 21st century, but immuno-oncology has revolutionized its treatment. Strategies based on the use of therapeutic monoclonal antibodies (mAbs) directed against immune checkpoint inhibitors^1–3^, or cytotoxic mAbs against specific tumor antigens have proved particularly effective^4,5^. Cytotoxic mAbs have been engineered to improve their therapeutic efficacy through the Fc-mediated immune effector function or by using antibody drug conjugates (ADCs). ADCs combine the specificity of a monoclonal antibody (mAb) with the toxicity of a cytotoxic payload^6^. ADCs allow a maximal anti-tumor efficacy via the specific targeting of the cytotoxic agent to tumor cells and minimize toxicity against healthy tissues^6^. An ADC is constituted by a mAb that is associated to a cytotoxic drug by a linker. The mAb must be as specific as possible for a cell surface tumor-associated antigen or overexpressed by tumor cells, in order to spare healthy tissue. Also, the targeted tumor antigen has to be internalized together with the ADC to deliver the cytotoxic agent inside the tumor cell. Another constraint of the ADC is the linker that bind the mAb to the cytotoxic agent. The linker can be cleavable or non-cleavable, meaning that the cytotoxic agent is released by a low pH or under reducing environment or require proteolytic degradation. In any cases, the release of the cytotoxic agent in the circulation must be avoided to prevent tissue damage. Conjugation of the linker to the mAb must not alter the binding specificity and affinity of the mAb. Finally, the cytotoxic agent coupled to the mAb can either disrupt microtubules or damage DNA, such as the pyrrolobenzodiazepines (PBD), both first and second class of ADC eventually inducing mechanisms causing cell death. Originally discovered in Streptomyces species, pyrrolobenzodiazepine (PBDs) are based on naturally occurring antitumour antibiotics that bind to the DNA minor groove. The dimerization of two PBD units allows to crosslink DNA by binding to the N2 position of guanine on opposing strands of DNA^7^. The rationally designed pyrrolobenzodiazepine (PBD) dimers emerged around ten years ago as a new class of drug for antibody-drug conjugates (ADC). Different PBD payloads including talirine and tesirine have been developed and evaluated clinically^7,8^. Talirine (SGD-1882) is highly hydrophobic with limited therapeutic index. All talirine-ADCs were subsequently discontinued because of high clinical toxicity^7,9^. Tesirine (SG3249) was designed to combine potent antitumor activity with desirable physicochemical properties such as favorable hydrophobicity and improved conjugation characteristics^7,10^. Recently Loncas-tuximab tesirine was approved under accelerated approval for the treatment of adult patients with relapsed or refractory large B-cell lymphoma after two or more lines of systemic therapy. Camidanlumab tesirine shows promising clinical trial results in the treatment of Hodgkin lymphoma. ADC design is very important to overcome toxicities and optimize therapeutic index. We developed site-specific technology based on bacterial transglutaminase (BTG) conjugation of derivative linker-drug to the glutamine at positions 295 and 297 thereby yielding homogeneous ADCs. As previously described these homogeneous ADCs display improved pharmacokinetics and better therapeutic indexes^11^. Classical dipeptide lysosomal-cleavable linker with high stability valine-alanine (VA) was selected^12^.

The number of approved ADCs has doubled in the last three years, reflecting the potential of this powerful tool to fight cancers^6^. Various tumor antigens have been targeted in this context. The most appropriate targets are accessible hematologicaltumor cell surface molecules (e.g. CD22, CD79b, BCMA, CD30, CD33), or highly expressed solid tumor antigens (e.g. Her-2, Nectin-4, Trop-2). Many targets have been explored in clinical development using the ADC approach and 11 of them have been approved. Targets with limited expression in normal tissues, which are well internalized and play a role in tumor biology (growth, metastasis or resistance), are potentially great assets for ensuring a positive efficacy/toxicity balance for this approach^13^.

Major histocompatibility complex (MHC) class I-related chain A and B polypeptides (MICA and MICB) are cell surface molecules with low levels of expression at steady state. However, they can be induced by cellular stresses, such as infections and tumor transformation^14–20^. The induction of their expression is tightly controlled by E2F, the DNA damage response and p53^21–24^. Together with members of the UL16-binding protein (ULBP) family, MICA and MICB act as ligands of the human NKG2D immunoreceptor, which is expressed on the surface of T cells (e.g. CD8+ T cells, γδ T cells) and natural killer (NK) cells^25–27^. Following ligand binding, NKG2D directly delivers activating signals promoting cytotoxicity and cytokine production^28^. MICA and MICB are frequently associated with epithelial tumors, induced by microbial infections, and is aberrantly expressed in certain autoimmune disease lesions^16,17,20,29^. MICA expression is also associated with hematological malignancies including leukemia and multiple myeloma^30–32^. MICA has a structure resembling the protein fold of MHC class I molecules, with an α1-α2 platform domain and a membrane-proximal Ig-like α3 domain^33^. Our objective was to develop an efficient therapeutic tool targeting MICA and MICB in oncological indications. We generated a group of cytotoxic mAbs targeting the products of the MICA and MICB alleles. The limited efficiency of these mAbs in models of solid tumors in vivo was the rationale to develop a novel ADC comprising the human anti-MICA/B Ab conjugated to highly potent (PBD) toxin with an intracellular cleavable linker using site-specific bacterial transglutaminase (BTG) technology yielding homogeneous ADCs. As previously described, these homogeneous ADCs display improved pharmacokinetics and better therapeutic indexes^11^. Preclinical efficacy and preliminary toxicity results validate MICA/B as an attractive therapeutic target for ADC approach in multiple solid tumors.

## Results

### MICA/B protein levels are low in healthy tissues and high at the tumor bed

**Figure 1:**
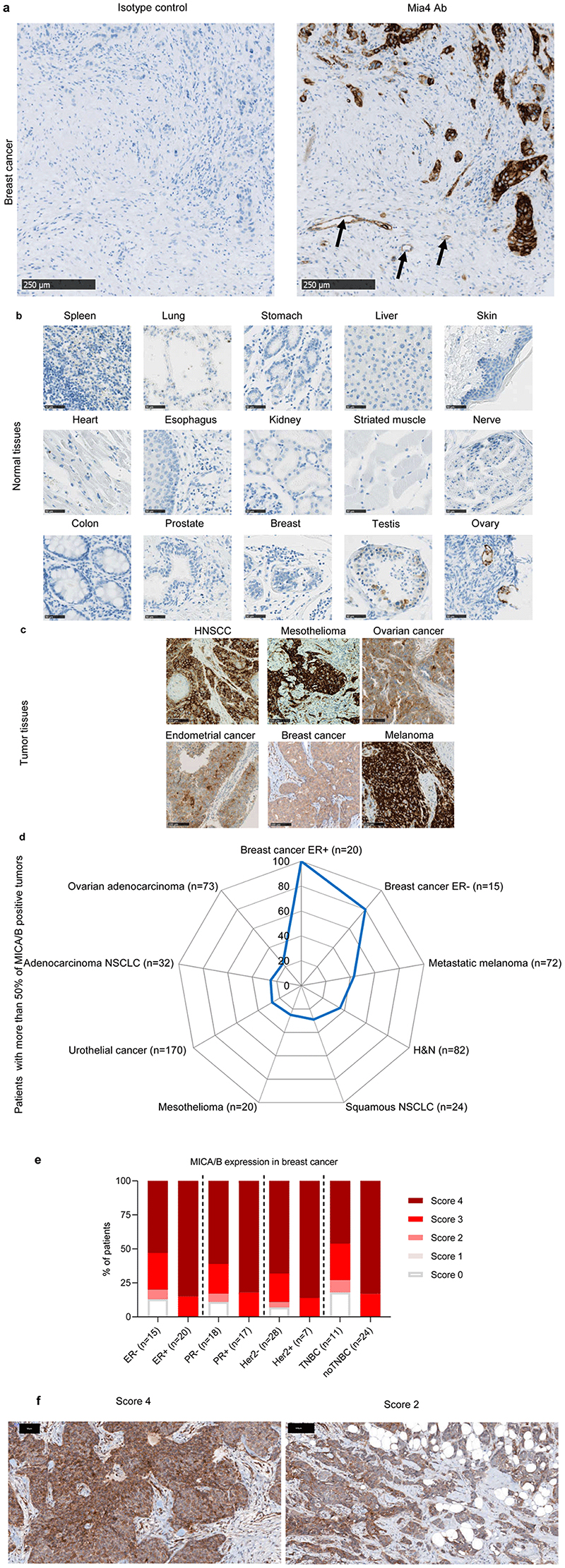
MICA and MICB expression are restricted to tumor tissues. (**a**) Two breast cancer slides were stained with Mia4 mAb developed by Innate Pharma or the isotype control (IC). MICA/MICB staining was positive only with Mia4 Ab compared to IC. Arrows indicate MICA/B-positive tumor-endothelium. Scale bars: 250 μm. (**b**–**c**) Representative immunohistochemical staining for MICA and MICB on 15 out of 32 tissues from three healthy donors (**b**) and on biopsy specimens from head and neck squamous cell carcinoma (HNSCC), mesothelioma, ovarian cancer, endometrial cancer, breast cancer and melanoma (**c**). Scale bars are indicated. (**d**) Percentage of patients with 50% of MICA/B-positive tumor cells on biopsy specimens from primary breast cancer estrogen receptor-positive (ER+) (*n*=20), breast cancer estrogen receptor-negative (ER-) (*n*=15), metastatic melanoma (*n*=72), HNSCC (*n*=82), squamous non-small-cell lung carcinoma (NSCLC) (*n*=24), mesothelioma (*n*=20), urothelial tumors (*n*=170), adenocarcinoma NSCLC (*n*=32), ovarian adenocarcinoma (*n*=73). (**e**–**f**) Different subtypes of breast cancer were scored based on proportion of MICA/MICB positive cells (score 0 (no MICA/MICB expression), score 1 (< 10%), score 2 (10% to 50%) score 3 (51 to 80%) and score 4 (>80%) tumor cells expressing MICA/MICB). (**e**) Patient proportions with different scores presented as histograms. (**f**) Two representative slides with scores 4 and 2. Strong cell surface MICA/MICB staining at different score. The black scale bars correspond to 50 and 100 μm.

We started by analyzing MICA/MICB protein levels in a large series of healthy tissues and tumors, as the quantification of MICA/MICB mRNA is not predictive of the amounts of the corresponding proteins expressed at the cell surface, due to the complex processes regulating these transcripts^34,35^. We therefore developed a novel mAb directed against both MICA and MICB, named Mia4, which specifically stained the MICA and MICB proteins on formalin-fixed, paraffin-embedded tumor tissue sections (Figure 1a). Healthy tissues were barely stained with the anti-MICA/B Mia4 mAb, including organs with high levels of blood flow, exposure, and clearance: the skin, liver, lung and all vital organs, heart and spleen. Only limited expression was detected on the testis and ovary (Figure 1b). By contrast, tumor biopsy specimens from patients with head and neck squamous cell carcinoma (HNSCC), mesothelioma, ovarian, endometrial cancers, breast and melanoma were stained with the anti-MICA/B Mia4 mAb, revealing high levels of MICA/B expression in tumors (Figure 1c). Importantly, MICA/B expression was observed at the membrane of tumor cells, in addition to intracytoplasmic expression (Figure 1a and 1b). Indeed, 20 to 100% of biopsy specimens from patients with ovarian adenocarcinoma, breast cancer (estrogen receptor-positive (ER^+^) and -negative (ER^-^)), metastatic melanoma, head and neck (H&N), non-small cell lung cancer (NSCLC, squamous and adenocarcinoma), mesothelioma and urothelial cancer, scored with more than 50% of cells positive for MICA/B expression (Figure 1d). Breast cancer tumor biopsies were scored based on the percentage of tumor cells expressing MICA (Figure 1e). Important membranous MICA/MICB staining was observed in tumor biopsies at various scores (Figure 1f). These results confirm the identification of MICA/B as an interesting candidate tumor antigen.

### MICAB1, a pan-allelic antibody, promotes *in vitro* antibody-dependent cellular cytotoxicity (ADCC) and phagocytosis (ADCP) and *in vivo* antitumor function

**Figure 2:**
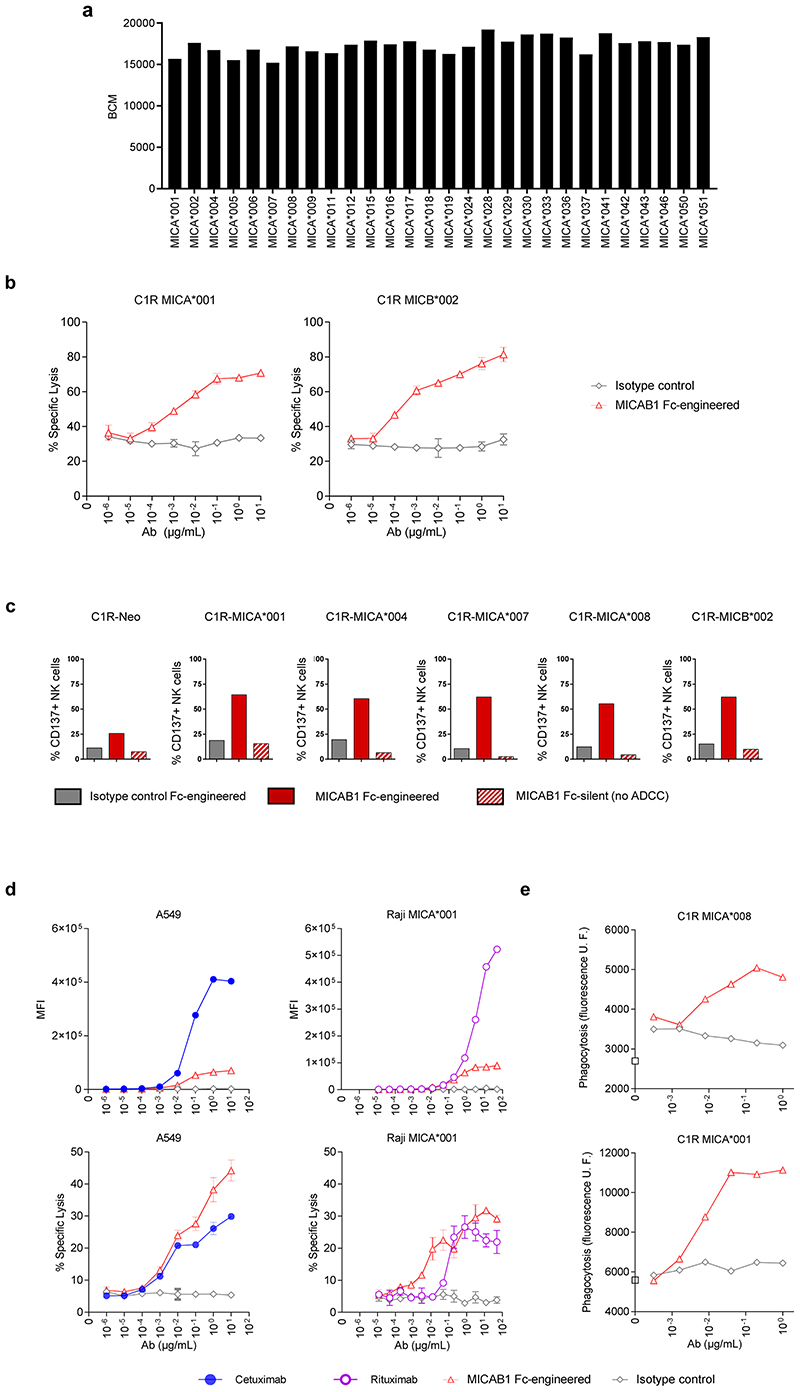
MICAB1, a pan-allelic antibody, mediates potent antibody-dependent cellular cytotoxicity (ADCC) and antibody-dependent cellular phagocytosis (ADCP). (**a**) Luminex assay characterizing MICAB1 monoclonal antibody (mAb) binding to 28 different alleles of MICA. Results are presented as the median fluorescence intensity corrected for the background control. Representative data from two independent experiments are shown. (**b**) Peripheral blood mononuclear cells (PBMCs) from healthy donors were incubated with ^51^chromium-loaded C1R tumor cell line expressing MICA*001 or MICB*002 in the presence of a range of doses of MICAB1 Fc-engineered or isotype control mAb. After four hours, ^51^chromium levels in the supernatants were determined. The data shown are representative of two independent experiments. Mean of percentage of lysis (n=3) and SD are shown. The percentage of lysis is significantly different between the MICAB1 Fc-engineered antibody and the isotype control for C1R MICA001 (p-value = 9.6e-08) and C1R MICB002 (p-value = 2.4e-09). The interaction between the antibody and its concentration is also significant in both experiments (p-values = 0.018 and 0.14, respectively). (**c**) C1R cells expressing different alleles of MICA were incubated with PBMCs from healthy donors and 10 μg/ml of MICAB1-Fc-engineered, MICAB1-Fc silent or isotype control Fc-engineered mAb for 24 hours. The data shown are the frequencies of natural killer (NK) cells expressing CD137 among total NK cells and are representative of three independent experiments (Friedman test p-value = 0.0007). Pairwise comparisons conclude that the percent of CD137+ NK cells obtained with MICAB1 Fc-engineered is significantly different from those obtained with all other antibodies (adjusted p-values =0.043). (**d**) PBMCs from healthy donors were incubated with ^51^chromium-loaded A549 cells naturally expressing MICA *001:12/*004 and MICB *002/*007 or Raji-MICA*001 in the presence of a range of doses of MICAB1 Fc-engineered or isotype control, cetuximab or rituximab. Upper panels: MFI of mAb binding at the cell surface (n=1) revealed with a secondary Ab. Lower panels: After four hours, ^51^chromium levels were determined in the supernatants. The data shown are representative of two independent experiments. Mean of percentage of lysis (*n*=3) and SD are shown. Antibody, its concentration and the interaction between both have a significant effect on the percentage of lysis in A549 (p-value = 4.9e-05 for antibody and 0.00016 for interaction) and in Raji-MICA*001 (p-value = 9.7e-09 for antibody and 0.01 for interaction). Pairwise comparisons conclude to a significant difference between MICAB1 Fc-engineered and the isotype control (p-value=1.3e-05 in A549 and 1.7e-09 in Raji-MICA*001) or Rituximab (p-value = 0.03) but not with Cetuximab (p-value=0.14). (**e**), C1R cells expressing MICA*001 or MICA*008 were loaded with PKH67 and added to macrophages in the presence of a range of doses of MICAB1 Fc-engineered or isotype control mAb. The cells were incubated for two hours at 37°C. Macrophage intracellular fluorescence intensity is shown (in relative units) as a function of antibody concentration. Data of one representative donor are shown.

For the targeting of MICA and MICB in cancer patients, we generated anti-MICA and MICB antibodies, by immunizing mice with recombinant soluble human MICA-Fc recombinant protein or a range of human C1R cells negative for endogenous MICA and MICB expression and engineered to express MICA alleles (Table 1). MICAB1 was selected based on its binding capacity to the products of 28 different alleles of MICA^36^ with Luminex technology, indicating that its epitope binding site was not affected by MICA and MICB allele polymorphisms (Figure 2a).

We then evaluated the ability of MICAB1 with Fc optimization to promote peripheral blood mononuclear cell (PBMC)-mediated ADCC towards human C1R cells expressing the MICA*001 or MICB*002 allele. MICAB1 mediated strong ADCC by resting PBMCs (Figure 2b). Similarly, Fc-engineered MICAB1 induced primary human NK cell activation in cocultures of PBMCs with human C1R cells expressing different alleles MICA or MICB, as shown by the induction of the cell surface activation marker CD137, whereas Fc-silent MICAB1 did not (Figure 2c). Epidermal growth factor receptor (EGF-R), CD20 and MICA and MICB were expressed on the surface of A549 lung cancer cells and Raji B-cell lymphoma cells (Figure 2d, upper panels). MICAB1, targeting MICA and MICB, promoted an efficient tumor target cell lysis by PBMCs when compared to the anti-EGF-R mAb cetuximab and the anti-CD20 mAb rituximab, which are widely used in clinical practice (Figure 2d, lower panels). MICAB1 also promoted the phagocytosis of MICA-expressing C1R target cells by monocyte-derived macrophages *in vitro* (Figure 2e). Thus, MICAB1 efficiently promotes the ADCC and ADCP of MICA-expressing tumor target cells *in vitro*.

**Figure 3:**
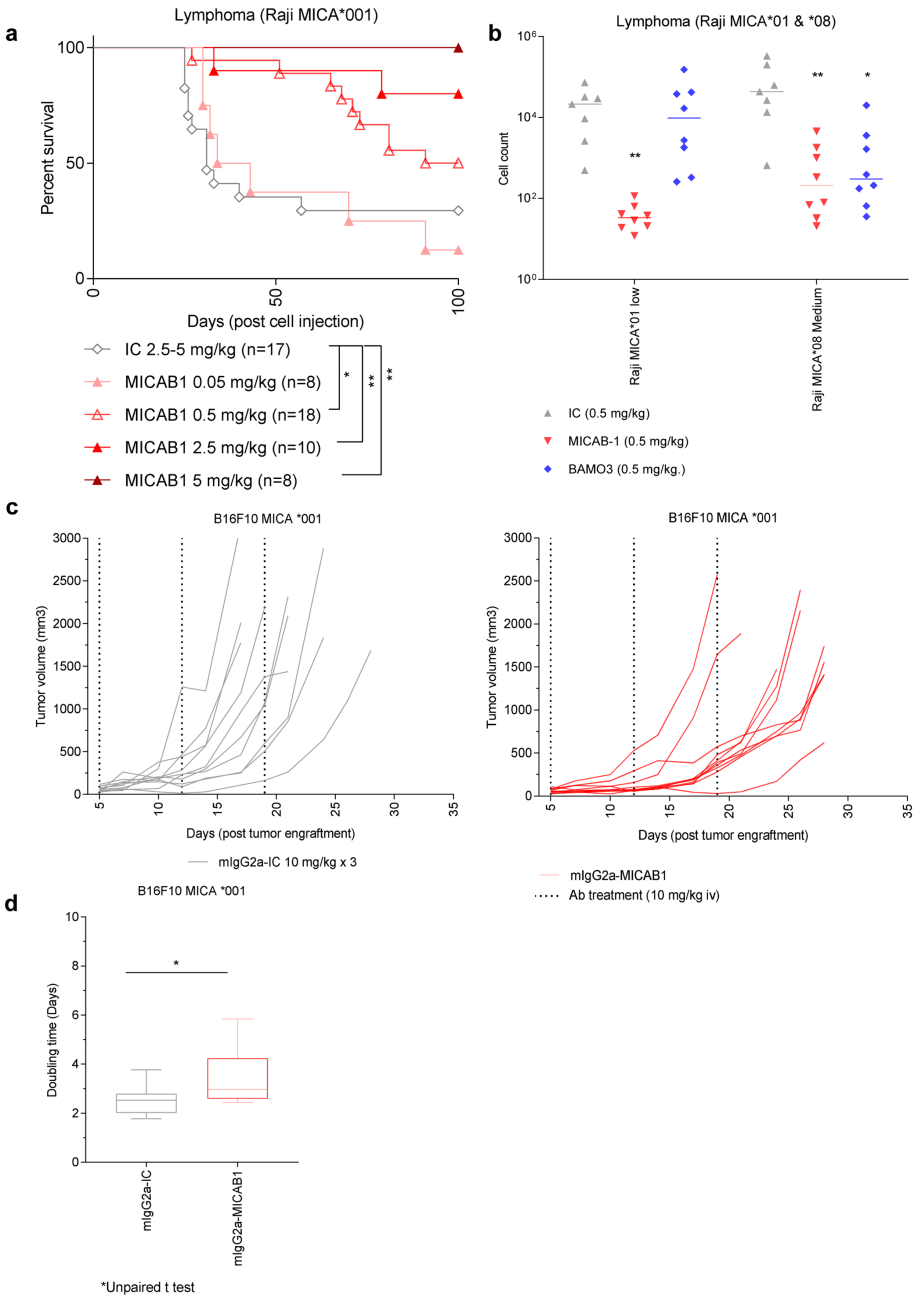
MICAB1 Fc-engineered prevents tumor growth but shows moderate therapeutic effect. (**a**) Raji B-cell lymphoma cells expressing the MICA*001 allele were injected intravenously into NOD-SCID immunodeficient mice simultaneously treated once with a range of doses of MICAB1 (0.05, 0.5, 2.5 or 5 mg/Kg, n=8 to 18, pool of two independent experiments) or isotype control monoclonal antibody (mAb) (2.5 or 5 mg/kg; n=17). Kaplan–Meier curves of mouse survival are shown. Pairwise comparison by Log-rank Mantel–Cox test was conducted between isotype control and MICAB1 0.05 mg/kg (p-value = ns), 0.5 mg/kg (p-value = 0.04), 2.5 mg/kg (p-value = 0.02) and 5 mg/kg (p-value = 0.009). There is a significant difference of survival between MICAB1 0.5 mg/kg and 5 mg/kg (p-value = 0.04). (**b**) Raji B-cell lymphoma cells expressing the MICA*001 or MICA*008 allele at low and medium levels were injected intraperitoneally into NOD-SCID immunodeficient mice simultaneously treated once with 0.5 mg/kg of body weight of MICAB1-Fc-engineered mAb or BAMO3 mAb or isotype control mAb. Tumor cell counts in peritoneal cavity lavage 24 hours later are shown. n=6-8 per group. Kruskal-Wallis test, p-value = 0.001. Tumor cell counts obtained with MICAB1 are significantly different from IC (p-value = 0.0017) but not from BAMO3 (p-value > 0.05). (**c**) B16-F10 MICA*001 were engrafted s.c. in MICAgen mice. Five days later, mice bearing tumors were randomized into two groups (n=10/group) and treated weekly for three weeks with 10 mg/kg of either MICAB1-mIgG2a or isotype control mAb. Tumor volumes per mouse over time are shown. The interaction antibody:time is significant (analysis of variance (ANOVA) on linear mixed-effects model (lmer) p-value = 0.0004), meaning that the tumor growth across time is different between antibodies. The pairwise difference of least squares mean for the factor antibody is statistically significant (p-value = 0.046). (**d**) Doubling time of tumor volumes from (**c**) are shown. Unpaired Wilcoxon test, p = 0.06.

We then assessed the *in vivo* efficacy of MICAB1. We used a tumor model consisting of NOD-SCID mice bearing invasive MICA-expressing B-cell lymphomas, resulting from the intravenous (i.v.) injection of Raji-MICA*001 tumor cells (Figure 3a). These mice were simultaneously treated with a range of doses of MICAB1 Fc-engineered mAb. We found that MICAB1 Fc-engineered mAb treatment was effective over a range of doses, with 50% and 100% of mice surviving at doses of 0.5 and 5 mg mAb/kg of body weight, respectively, versus 30% of the mice treated with the control mAb at a dose of 5 mg/kg (Figure 3a). MICAB1 Fc-engineered mAb treatment also decreased the numbers of Raji-MICA*001 and MICA*008 tumor cells (exhibiting low and medium levels of surface MICA, respectively), recovered from the peritoneal cavity of NOD-SCID mice, relative to control mAb-treated mice (Figure 3b). In this model MICAB1 Fc-engineered mAb was more potent than commercial anti-MICA/B mAb BAMO3 at controlling tumor cells with low level of MICA (Figure 3b).

We then evaluated the therapeutic effect of MICAB1 mIgG2a mAb on tumor models based on subcutaneous (s.c.) injection of B16F10-MICA*001 in immunocompetent MICAgen transgenic mice^34^ (Figure 3c and d). Beneficial effects of MICAB1 were moderate for the treatment of solid tumor models, compared to intravenous or intraperitoneal tumor models.

### Generation and characterization of MICAB1 PBD1G-ADCs

**Figure 4:**
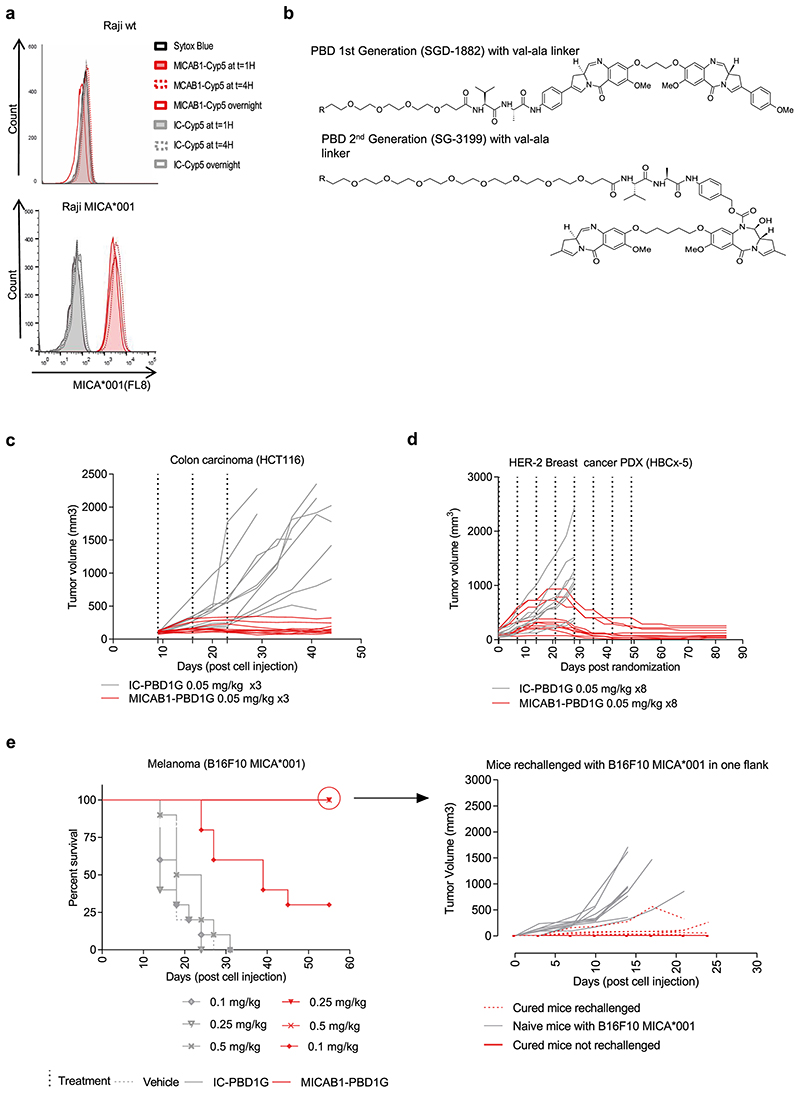
*In vitro* internalization and *in vivo* efficacy of MICAB1 PBD1G antibody-drug conjugates (ADCs). (**a**) Raji cells and Raji cells expressing the MICA*001 allele were incubated for one, four or 16 hours (overnight) with MICAB1 or isotype control monoclonal antibodies (mAbs) coupled to a pH-sensitive cyanine dye (Cyp5). Fluorescence-activated cell sorting (FACS) profiles showing dye fluorescence upon anti-MICA mAb internalization are shown. Representative data from two independent experiments are shown. (**b**) Structures of PBD1G: PEG(4)-val-ala-SGD-1882; PBD2G: PEG(8)-val-ala-SG3199. (**c**) HCT-116 cells were engrafted, subcutaneously (s.c.), into nude mice. 10 days later, tumor-bearing mice were randomized to two groups and treated once weekly, for three consecutive weeks, with 0.05 mg/kg MICAB1 PBD1G-ADCs or IC-PBD1G. Tumor growth was followed over time (n=10 per group). The tumor growth is significantly different between antibodies (analysis of variance (ANOVA) on linear mixed-effects model (lmer) p-value = 0.0002). The interaction antibody:time is also significant (ANOVA on lmer p-value = 1.2e-23). (**d**) HER2 HBCx-5 fragments were engrafted into SHO mice. 35 days later, tumor-bearing mice were treated once weekly for eight consecutive weeks, with 0.05 mg/kg MICAB1 PBD1G-ADCs or IC-PBD1G. Tumor growth was followed over time (n=10 per group). After day 28, no mice survived in the control group: we cannot perform the linear mixed model after that day. Considering time until day 28, antibody is not a significant factor but its interaction with time is (ANOVA on lmer p-value = 2.2e-19). (**e**) B16F10 cells expressing MICA*001 were engrafted, s.c., into C57BL/6 mice. Four days later, when tumors reached 150 mm^3^, mice were randomized to six groups and treated once weekly for three consecutive weeks with 0.1 mg/kg, 0.25 mg/kg or 0.5 mg/kg MICAB1 PBD1G-ADCs or IC-PBD1G. Kaplan–Meier curves show mouse survival (n=12 per group). Pairwise comparison by Log-rank Mantel–Cox test between MICAB1 0.5 mg/kg or 0.25 mg/kg with MICAB1 0.1 mg/kg (p-value = 0.002), between MICAB1 0.5 mg/kg or 0.25 mg/kg with isotype controls (p-value = 9.4e-06) and between MICAB1 0.1 mg/kg with isotype controls (p-value = 0.0002) is shown. Right panels: Cured mice (n=9) and naïve mice (n=10) were re-challenged s.c. with B16F10 expressing MICA*001 on one flank. Some cured mice (n=4) were not re-challenged and monitored for eventual tumor regrowth. Tumor growth over time is shown. Tumor growth is significantly different between naive mice rechallenged with B16F10 MICA001 vs cured mice rechallenged with B16F10 MICA001 (p-value = 5.7e-06). Representative data from two independent experiments for HCT116 and B16F10 MICA*001 and one experiment for patient-derived xenograft (PDX) are shown.

We then aimed to improve the therapeutic effect of the MICAB1 mAb in solid tumors, using a MICAB1 Fc-silent antibody conjugated to potent cytotoxic payloads. We checked that MICA was internalized after binding to the MICAB1 mAb. The binding of the MICAB1 Fc-silent mAb coupled to a pH-sensitive dye on MICA-transfected Raji cells led to an increase in the intensity of the fluorescence signal during the first hour, consistent with rapid internalization (Figure 4a). For the payloads, we focused on the highly potent pyrrolobenzodi-azepine family (PBD), to maximize the potential efficacy of MICAB1-ADCs against a broad range of tumors with various levels of MICA and MICB expression^37^. The MICAB1 Fc-silent mAb was coupled to a first-generation PBD (PBD1G) with BTG technology^8^, which has been reported to limit off-target toxicity, at a drug-antibody ratio (DAR) of 2, via a intracellular valine-alanine cleavable peptide linker (Figure 4b). *In vivo*, the MICAB1-ADCs had a strong effect in a xenogenic colon carcinoma model HCT-116, in nude mice, at doses as low as 0.05 mg/kg, administered once weekly for three weeks, as demonstrated by comparison to an isotype control (Figure 4c).

We then tested the MICAB1-ADCs in patient-derived xenograft (PDX) models, which present the obvious advantage of originating from cancer patients, and hence may better reflect human tumor physiology and preserve the heterogeneity of the original tumors. We performed immunohistochemical staining (IHC) to assess MICA/B expression in a panel of 146 PDXs corresponding to different types of human cancer (breast, lung and colon cancer). The distribution of MICA expression in PDXs matched that observed in patients (Figure 1 and Table 2). MICAB1 PBD1G-ADCs had a curative effect in a PDX model of HER-2^+^ breast cancer, at doses as low as 0.05 mg/kg once weekly for eight weeks, with a complete remission rate of 100% on day 85 (Figure 4d). MICAB1 PBD1G-ADCs also increased the survival of C57BL/6 mice receiving injections of MICA-transfected B16F10 melanoma cells, and the cured mice developed long-term protective immunity when rechallenged with B16F10 MICA*001 (Figure 4e). Thus, MICAB1 PBD1G-ADCs were reactive against several xenogenic and syngeneic tumor models *in vivo*.

### MICAB1 PBD2G ADCs inhibit tumor growth *in vivo*

The PBD1G-ADCs have raised safety issues in several clinical trials^9,33^. We therefore coupled the MICAB1 mAb to a second-generation PBD (PBD2G), less toxic for the target cells^8^ and with a better therapeutic index.

**Figure 5:**
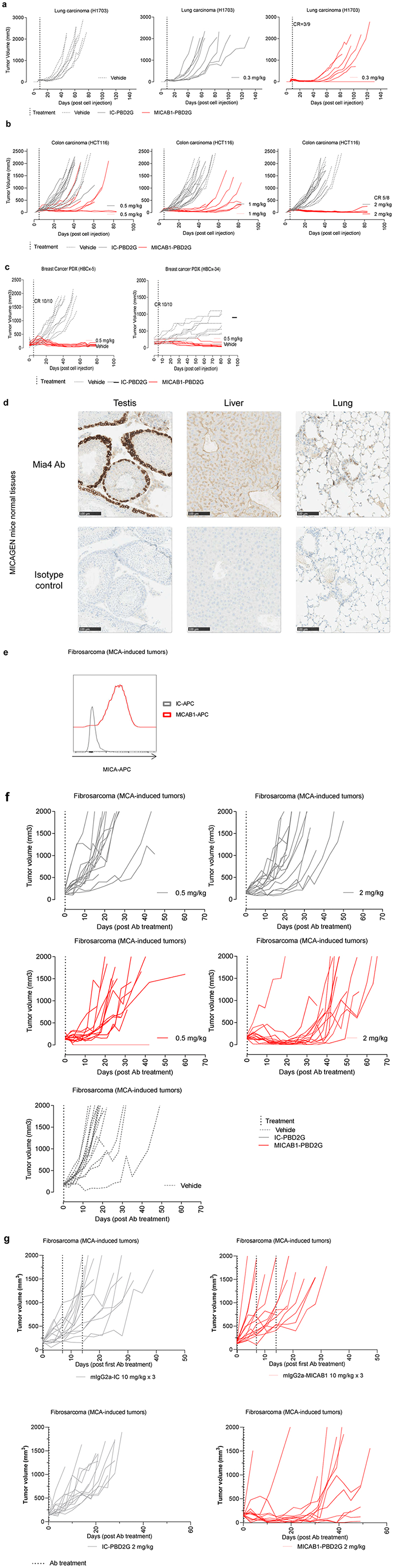
Therapeutic benefit of MICAB1 antibody-drug conjugate (ADC) *in vivo* in mouse preclinical models. (**a**) Lung adenocarcinoma H1703 cells were engrafted subcutaneously (s.c.) in nude mice. Nine days later, tumor-bearing mice were randomized to three groups and treated once with 0.3 mg/kg MICAB1 PBD2G-ADCs, IC-PBD2G or vehicle. Tumor volumes per mouse over time are shown (*n*=9 per group). Tumor growth over time is significantly different between MICAB1 PBD2G-ADCs and vehicle or IC-PBD2G (analysis of variance (ANOVA) on linear mixed-effects model (lmer) and pairwise comparisons, p-values <0.05). (**b**) HCT-116 cells were engrafted s.c. in nude mice five to six days later, tumor-bearing mice were randomized to six groups and treated once with 0.5, 1 or 2 mg/kg MICAB1 PBD2G-ADCs, IC-PBD2G or vehicle. Tumor growth was followed over time (*n*=8-9 per group). Representative data from two independent experiments are shown. For all three concentrations, MICAB1 PBD2G-ADC’s effect on tumor growth is significantly different from the vehicle and from the IC-PBD2G. (ANOVA on lmer and pairwise comparisons, p-values <0.05). (**c**) HER2 HBCx-5 or luminal HBCx-34 patient-derived xenografts (PDX) were engrafted into nude mice. 35 to 38 days later, tumor-bearing mice were treated once with 0.5 mg/kg MICAB1 PBD2G-ADCs or vehicle. Tumor volumes per mouse over time are shown (*n*=10 per group). MICAB1 PBD2G-ADCs has a significant impact on tumor growth (ANOVA on lmer p-value for the interaction between antibody and time <0.05). (**d**) Formalin-fixed, paraffin-embedded FFPE tissues slides from testis, liver and lung were stained with the anti-MICA/B Mia4 mAb developed by Innate Pharma or the isotype control at 2 μg/ml, membranous and/or cytoplasmic staining was observed in several tissues. (**e**) Methylcholanthrene (MCA) tumors were dissociated and stained with MICAB1 or IC-APC mAb. MICA cell surface expression was determined by flow cytometry. (**f**) MICAgen mice were treated s.c. with 100 μg MCA. Around 100 days later, mice bearing fibrosarcomas were randomized into five groups and treated once with vehicle or 0.5 or 2 mg/kg MICAB1 PBD2G-ADCs or IC-PBD2G or vehicle. Tumor volumes per mouse over time are shown (n=15 per group). MICAB1 PBD2G-ADCs had a significant impact on tumor growth with both concentrations (ANOVA on lmer p-value for the interaction between antibody and time < 0.05). (**g**) MICAgen mice were injected s.c. with MCA. Around 100 days later, mice bearing fibrosarcomas were randomized into four groups. Two groups were treated once with 2 mg/kg of MICAB1 PBD2G-ADCs or IC-PBD2G and two groups were treated once a week for three weeks with 10 mg/kg of mIgG2a-MICAB1 or mIgG2a-IC. Tumor volumes per mouse over time are shown (n=13 or 14 mice per group). One experiment was performed. Interaction between antibody and time is significant (ANOVA on lmer p-value = 2.6e-14). Pairwise comparisons show significant differences between MICAB1-PBD2G and IC-PBD2G (p-value = 0.0006), MICAB1-PBD2G and mIgG2a-MICAB1 (p-value = 1e-05).). For PDX models, one experiment was performed.

The therapeutic effect of MICAB1 PBD2G-ADCs was then evaluated *in vivo*, in immunodeficient NOD-SCID mice into which H1703 lung carcinoma cells had been injected. MICAB1 PBD2G-ADC treatment was effective at a single low dose of 0.3 mg/kg, with one third of the mice displaying complete remission and a significant delay of apparent tumor growth, by ~40 to 60 days relative to the control mAb (Figure 5a). In a second xenogenic model, nude mice engrafted with HCT116 colon carcinoma were treated with MICAB1 PDB2G-ADCs at doses of 0.5, 1 and 2 mg/kg, which delayed apparent tumor growth, by ~20, 50 and 80 days, respectively, relative to the isotype control PBD2G conjugate (Figure 5b). We then assessed two breast cancer PDXs, HER-2^high^ (HBCx-5) and HER-2^low^ luminal (HBCx-34)^38^, with high and medium levels of MICA expression, respectively, in nude mice. Single injection of MICAB1 PDB2G-ADC at low dose of 0.5 mg/kg effectively controlled tumor growth in both breast cancer models with 100% of complete remission on day 85 (Figure 5c).

Although the transgenic MICAgen mouse represents a valuable model to study MICA *in vivo*^34^, IHC analyses of tissues from these mice revealed MICA expression in several organs, whereas no such expression was observed in human organs (Table 3 and Figure 5d). Nevertheless, we established a cancer model in MICAgen mice, by injecting a carcinogen, methylcholanthrene (MCA), which presents the advantage of reconstituting real carcinogenesis and the associated tumor microenvironment. Importantly, MCA-induced fibrosarcomas displayed cell-surface MICA expression (Figure 5e). The therapeutic effect of MICAB1 PBD2G-ADCs was then evaluated in mice bearing tumors induced by MCA injection (Figure 5f). MICAB1 PBD2G-ADC treatment displayed signs of activity even for a single dose of 0.5 mg/kg, relative to the control group treated with IC-PBD2G at the same dose. A single injection of 2 mg/kg MICAB1 PBD2G-ADC delayed tumor growth considerably by 20 days. Of note, no therapeutic effect was observed with the MICAB1 mAb in MCA-induced fibrosarcomas bearing MICAgen mice even at optimal doses of 10 mg/ml once weekly for three weeks (Figure 5g).

Altogether, these results showed that MICAB1-PBD2G-ADC are efficient to treat solid tumors with 100% of complete remission on day 85 in two PDXs treated mice and are well tolerated in MICAgen humanized mice.

## Discussion

MICA and MICB are tightly regulated stress-induced proteins that trigger the immune system by binding to the activating receptor NKG2D on cytotoxic lymphocytes. Targeting the NKG2D-MICA/B axis is a very attractive immunotherapeutic approach. Conceptually different ways of targeting MICA are currently being investigated, including bispecific T-cell engagers B2-OKT3 (MICA, CD3), bispecific antibodies, such as JZC01 (MICA, VEGFR2)^39^, 2A9-MICA (MICA, BCMA)^40^, cytotoxic anti-α3 domain–specific mAbs for inducing ADCC^41,42^ and CAR-NK cells. Targeting MICA/B using an ADC approach is, to our knowledge, totally novel.

It was previously reported that MICA and MICB are predominantly expressed intracellularly in tumor and normal tissue using commercially available antibodies directed against MICA α1/2 or α3 domains^36^. In contrast, our results indicate that limited MICA expression was detected on healthy tissues in comparison with an important expression in tumors in large cohorts of patients, that includes both membranous and intracellular expression, as for other solid tumor antigens (Her-2, Nectin-4, TROP-2).

Using *in vitro* assays with fluorescent dye, we show here that MICAB1 induces a high level of MICA internalization, supporting the development of ADCs targeting MICA and MICB using intracellular cleavable linkers. We observed complete remission in most of the tumor models tested with the MICAB1-ADC approach. In addition, a strong vaccination effect was obtained with MICA-ADC in a syngeneic MICA-transfected melanoma model.

Our ADC technology includes an enzymatic conjugation method using BTG, facilitating the site-specific conjugation of the linker-toxin to the mAb, thereby making it possible to control the drug-antibody ratio. We coupled the MICAB1-Fc silent mAb to potent DNA binder payloads, to promote the killing of MICA/MICB-expressing tumor cells. ADCs carrying a new generation of PBDs are currently in clinical development. ADCT-402 (loncastuximab tesirine), an ADC against CD19 conjugated to a PBD-dimer toxin, has recently been approved for the treatment of diffuse large B-cell lymphoma and mantle cell lymphoma, on the basis of clinical results obtained in phase I and pivotal phase II clinical trials^43^. As the therapeutic index is lower for these potent payloads, good targets must display tumor-restricted expression. In addition, our technology, based on site-specific conjugation with an Fc silent mAb, improves the therapeutic index of ADCs^11^. The use of a genetically engineered mouse model expressing human MICA (MICAgen mice^34^), in conjunction with carcinogen-induced tumors, further validates the presence of a therapeutic window for our ADCs, without major toxicity, even in the presence of low levels of MICA on normal tissues.

Like other cell surface tumor antigens (Her-2, Nectin-4, CD30), MICA is subject to proteolytic cleavage^44–46^. The shedding of this protein generates soluble truncated MICA molecules including only the extracellular domain^42,45,47^. Given the levels of soluble MICA and MICB found in the serum of cancer patients (~100 pg/mL)^48^, and the concentrations of PBD2G-ADCs at doses currently used in clinical practice (~500 and 700 ng/mL), the efficacy of MICAB1 PBD2G-ADCs should not be affected by the presence of soluble MICA, but further investigations aiming at addressing this point will be needed.

Therefore, several approaches are proposed to harness antitumor immunity via the targeting of MICA/B. Our results are based on an array of *in vivo* models including MCA-induced fibrosarcoma in an immunocompetent ad hoc MICA-transgenic mouse model, and PDX. Importantly, efficacy of MICAB1-PBD2G at low dose was observed in two-breast cancer PDXs, including HBCx-34 which is resistant to another ADC targeting trastuzumab emtansine (KADCYLA), targeting the HER2 tumor antigen^38^. Thus, MICAB1-PBD2G appears a promising therapeutic tool for further clinical development. The next steps for the clinical development of MICA ADCs will include the assessment of their safety profile in non-human primates.

## Methods

### Antibodies

Cat# MICAB1, RRID:AB_2892111, Innate Pharma; Cat# MICAB1-mIgG2a, RRID:AB_2892224, Innate Pharma; Cat# MICAB1 Fc-silent RRID:AB_2892226; Innate Pharma; Cat# Mia4 mAb AB_2892227, Merk Serono; Cat# Erbitux, RRID: AB_2892606, ROCHE; Cat# Mabthera, AB_2892607 Innate Pharma; Cat# IC-mIgG2a RRID:AB_2892232, Innate Pharma; Cat# IC Fc-silent RRID:AB_2892233, Innate Pharma; Cat # Isotype control RRID:AB_2892234, Innate Pharma; Cat#MICAB1-PBD2G, AB_2892235, Innate Pharma; Cat#MICAB1-PBD1G, AB_2892236, Innate Pharma; Cat# IC-PBD2G AB_2892237, Innate Pharma; Cat#IC-PBD1G, AB_2892238, Innate Pharma; Cat# MICAB1-Cyp5, AB_2892239, Innate Pharma; Cat# IC-Cyp5 RRID:AB_2892241, Innate Pharma; Cat#MICAB1-APC, RRID:AB_2892242, Innate Pharma; Cat# IC-APC RRID:AB_2892243, BAMO3 monoclonal mouse anti-human alpha3 MICA/B antibody from Tubingen university.

### Cell lines

C1R and CIR transfected with MICA/B alleles (C1R MICA or C1R MICB) or ULBP cells were previously reported^32^^,^^45^. B16F10 were purchased (ATCC, cat. n. crl-6475tm) and transduced with different MICA and MICB alleles. Positive cells were sorted by flow cytometry and expanded in culture. Indicated cell lines were transfected by nucleofection using the 4D-NucleofectorTM system from Lonza (Program FF-120, SF solution). The day after transfection, the hygromycin selection was added to the nucleofected cells at 400 μg/ml. All other tumor cells were purchased from ATCC (H1703, cat. n. CRL5889, HCT116, cat. n. CCL-247, A375 cat. n. CRL-1619, A549 cat. n. CCL-185, BxPC-3 cat. n. CRL-1687) and were cultivated, as recommended by the supplier, in RPMI (cat. n. 31870025, Gibco) or DMEM (cat. n. 11966025, Gibco) supplemented with 10% FCS (Fetal Calf Serum, cat. n. 10270106, Gibco), with or without 1% L-Glutamine 200mM (cat. n. 25030123, Gibco), 1% Sodium Pyruvate 100mM (cat. n. 11360070, Gibco).

**Table:** 

Cell line	Characteristics
A549	Human lung cancer cells
B16F10	Mouse melanoma cells
C1R	Human B lymphoblast
H1703	Human lung carcinoma cells
HBCx-34	HER-2low luminal breast cancer patient-derived xenografts
HBCx-5	HER-2high breast cancer patient-derived xenografts
HCT-116	Human colon carcinoma cells
MCA-induced tumors	Methylcholanthrene induced tumors
Raji	Human B-cell lymphoma cells

### Mice

Ethical approval for Innate Pharma study was obtained from CEEA-IPH (CNREEA registration code CEEA – 070). Project authorization using animals for scientific purposes was provided by the French Ministry of Higher Education, Research and Innovation under the number APAFIS#25600. Approval number for the use of animals at Innate Pharma used for scientific purposes is C 13 055 30. MI-mAbs Authorization (Apafis#6653-2016072612069416 v6) was delivered by the French Minister of Enseignement supérieur, de la recherche et de l’innovation after approval by the Marseille Ethical Committee for Animal Experimentation (Comité National de Reflexion Ethique sur l’Expérimentation Animale no. 14). Mice were handled in accordance with national and European laws for laboratory animal welfare and experimentation (EEC Council Directive 2010/63/EU, September 2010). Xentech authorization to use animals in the CERFE facility was obtained by The Direction Dèpartementale de la Protection des Populations, Ministère de l’Agriculture et de l’Alimentation, France “Direction of the Veterinarian Services, Ministry of Agriculture and Food, France” (agreement No. D-91-228-107). All PDXs experiments were performed in accordance with French legislation concerning the protection of laboratory animals and in accordance with a currently valid license for experiments on vertebrate animals, issued by the French Ministry of Higher Education, Research and Innovation (#14073).

C57BL/6J and nude mice were reared at Janvier Laboratories and Charles River laboratories. Transgenic mice with a 23.4 kb human genomic fragment (MICAgen mice), comprising the entire huMICA*007 allele and expressing MICA in a strictly controlled manner, as recently described^34^ were obtained from Steinle lab. MICAgen mice are licensed for Innate Pharma experiments by Fred Hutch and Goethe University. Rj:NMRI-Foxn1 nu/nu, cat# SM-NMRNU-F mice were obtained from Janvier Laboratories. Athymic Nude - Foxn1nu mice from ENVIGO (Gannat, France) and Crl:SHO-Prkdcscid Hrhr SHO mice from Charles River Laboratories and used by Xentech for PDXs models. Mice were received in the SOPF experimental zone at least one week before the beginning of the experiments. All animal experiments were performed in accordance with the rules of the Innate Pharma ethics and animal welfare committees.

### Human primary cells

Peripheral blood samples from healthy donors were obtained from the Etablissement Francais du Sang (EFS, Marseille) under a written consent obtained from each volunteer by the EFS (transfer agreement #AC-2019-3428). Tumors tissues from patients were obtained at the time of surgical resection under approved protocol and written informed consent from each patient. The protocol was approved by local ethics and human investigations committee (IDRCB #2017-A00778-45) and by the Assistance Publique des Hôpitaux de Marseille (AP-HM).

### IHC

Formalin-fixed paraffin embedded blocks were sliced in 5 μm-thick sections and immunostainings performed on a Discovery Ultra or a Benchmark Ultra (Ventana). All samples except breast cancer tissues were stained on a Discovery Ultra automaton. After pre-treatment with cell conditioning 1, sections were incubated for 1 hour at 37°C with anti-MICA/B (clone Mia4, Innate Pharma) primary antibody or mouse IgG1 isotype control at 2 μg/mL (for staining on the Discovery Ultra) or at 6.6 μg/mL (for staining on the Benchmark Ultra). Then, signal amplification using the discovery Amp HQ kit (760-052) or the UltraView kit (760-500) was performed. After revelation with 3,3-diaminobenzidine, counterstaining with hematoxylin and bluing, sections were washed, dehydrated, cleared and coverslipped. Stained sections were finally scanned on a slide scanner (S60 Nanozoomer, Hamamatsu or a Pannoramic scan II, 3DHistech). Staining was interpreted and scored by trained pathologists that determined the MICA/B expression on the tumor cells. Samples with more than 1% of MICA/B positive tumor cell were considered MICA/B positive.

### Normal tissues used for IHC analysis

Panel of multiple organs of normal tissue microarray (cat. n. FDA999k, BioMax Us) were stained with Mia-4 as per the previously described IHC protocol. In total 32 types of normal human organs were analyzed including cerebrum, cerebellum, adrenal gland, ovary, pancreas, parathyroid gland, hypophysis, testis, thyroid gland, breast, spleen, tonsil, thymus gland, bone marrow, lung, heart, esophagus, stomach, small intestine, colon, liver, salivary gland, kidney, prostate, uterus, uterine cervix, skeletal muscle, skin, peripheral nerve, mesothelium, eye and larynx, each type taken from three normal human individuals, single core per case.

### Production and purification of antibodies

Antibodies were produced as previously described^11^.

The sequences encoding the light chain variable and heavy chain variable domains of the various antibodies were inserted into the SLX-192 expression vector in frame, it could be replaced with Gateway™ pDEST™26 Vector (Thermofisher 11809019) with the desired human or mouse constant regions. Expression vectors for the light chain and the heavy chain of each antibody (prepared as endotoxin-free midipreps) were used to co-transfect a CHO cell line (Thermofisher K1535). The cells were used to seed culture flasks at a density of 3 x 105 cells per mL and cultured in BalanceCD medium (Fujifilm). The supernatants were harvested after ten days and passed through a Stericup filter with 0.22 μm pores (Sartorius 180C2). Antibodies were purified with MabSelect PrismA beads (Cytiva 17549802), eluted with 0.1 M sodium citrate buffer at pH 4.5 and immediately neutralized with 1 M Tris pH 8.5. The proteins were then dialyzed overnight with PBS1X at 4°C and analyzed to check for the absence of aggregates and endotoxins. When the MICAB1-Fc engineered or Fc-silent antibody was used, isotype control exhibit the corresponding Fc portion in all cases.

### Production of MICAB1 Fc-silent having N297S mutation

Antibodies were produced as previously described^11^. Briefly, IPH43 Fc-silent was produced as chimeric antibodies bearing human IgG1 constant regions comprising an asparagine to serine substation at Kabat heavy chain residue 297 (N297S), thereby eliminating native N297-linked glycosylation. The antibody comprised an acceptor glutamine at amino acid residue 295 (Kabat EU numbering) of their heavy chains, such that the reactive linker was conjugated to residue Q295 on each heavy chain of the antibodies (each antibody has two conjugated moieties; DAR=2), and lacking significant effector function.

### Luminex assay

A LifeCodes LSA-MIC Luminex kit (Immucor, cat. n. 265300R) was used to determine the binding of MICAB1 and an isotypic control to the products of 28 different alleles of MICA, as recommended by the manufacturer and described elsewhere^36,49^.

### Binding of MICAB1 to cells expressing MICA

Cell staining was performed with the range of doses of MICAB1 or isotype control (IC) mAb indicated in the corresponding experiments, to monitor binding to MICA on cells. Various doses of antibody were incubated with the cells for 1 hour at 4°C. The cells were then washed and goat anti-human IgG (H+L)-PE secondary antibody (IM1626, BC) was added and the cells were incubated for a further 30 minutes at 4°C. The cells were washed and then acquired and analyzed on a FACS CANTO II (HTS) (BECTON DICKINSON (BD)) flow cytometer equipped with FACS Diva software, and analyzed with FlowJo X 10.0.7r2 software.

### ^51^Chromium release assay

Target cells were stained with 50 μCi of ^51^chromium (PERKIN ELMER, cat. no. NEZ030002MC) per million cells for 1 h at 37°C. 50 μL of antibody were loaded onto a 96-well plate, completed with 100 μL of ^51^chromium-loaded target cells and then 50 μL of NK or PBMC. The plates were then incubated for 4 h at 37°C, and 50 μL of supernatant was collected and transferred to a LumaPlate (Perkin Elmer, cat. no. 6006633). Plates were dried at 56°C and read with a TopCount (NXT™ Perkin Elmer) apparatus. A control condition without antibody was used to assess basal target cell lysis by NK or PBMC. In addition, a control without antibody and without NK or PBMC was used to determined spontaneous ^51^chromium release from the target cells. A control with 2% Triton X-100 on target cells was used to determine the maximal level of ^51^chromium release from target cells. Experiments were performed in duplicate or triplicate, depending on the number of effector cells obtained.

### ADCC assay

PBMCs were isolated from buffy coat samples (EFS: *Etablissement Français du Sang*, Marseille) by density gradient separation (Pancoll tube: PAN BIOTECH, cat. no. P04-60100). The PBMC preparation was then enriched in NK cells by negative selection with a human NK cell isolation kit and LS separation columns (MACS-Miltenyi, cat. no. 130-092-657) in accordance with the manufacturer’s instructions. PBMCs were used at and effector:target (E:T) cell ration of 100:1 or 200:1, whereas purified NK cells were used at an E:T ratio of 10:1. A range of antibody doses was added to the effector cells. ^51^Chromium-loaded target cells were added and classical chromium release assay performed.

### ADCP assay

Monocytes were isolated with the human Kit CD14 microbeads (Miltenyi, cat. no. 130-050-201), resuspended in macrophage medium (complete medium: RPMI-1640 containing 10% FCS, 2 mM L-glutamine, 1 mM sodium pyruvate and 1x NEAA supplemented with 20 ng/mL M-CSF and 1x penicillin/streptomycin solution) and differentiated for seven to nine days, with medium renewal every two to three days. The day before the ADCP experiment, monocyte-derived macrophages were used to seed a flat-bottomed 96-well plate (100 000 macrophages/well) in complete RPMI medium, incubated overnight at 37°C and starved from FCS for 2 h in RPMI without additives. Antibodies were diluted and added to the plates containing macrophages. PKH67 (SIGMA, cat. no. PKH67GL-1KT)-labeled target cells were added to macrophages with an E:T ratio of 1:4 (400 000 target cells /well). The plate was centrifuged for 2 min at 300 x g and incubated for 2 h at 37°C. After incubation, the supernatants containing non-phagocytosed target cells in suspension were discarded, and trypan blue (diluted 1/2 in PBS, 50 μL/well) was added to the plate and incubated with the cells for 1 minute to quench extracellular fluorescence. The trypan blue was then removed and the plate was analyzed on an EnSpire® Multimode Plate Reader (Perkin Elmer) to quantify residual PKH67 fluorescence from cells subjected to phagocytosis, corresponding to macrophage intracellular fluorescence.

### Cypher-5 internalization

MICA antibodies or IC were conjugated with CypHer5E Mono NHS Ester (Ge Healthcare, cat. no. PA15401), as recommended by the supplier. MICAB1-Cypher5 or IC-Cypher5 at 1 μg/mL was added to 50 000 Raji wt or Raji MICA*001 cells, and the mixture was incubated for 30 minutes, 1 h, 4 h or overnight at 37°C. Internalization was stopped by placing the plate on ice for 10 minutes. The cells were washed and resuspended in cold PBS containing the mortality marker Sytox blue (Thermo Fisher Scientific, cat. no. S34857) diluted 1/10000. Cells were acquired and analyzed on a FACS CANTO10 (BECTON DICKINSON (BD)) flow cytometer, with FACS Diva software.

### Conjugation of MICAB1 to first- or second-generation (1G or 2G) pyrrolobenzodiazepines

Conjugations were performed as previously described^11^. PBD1G was purchased from Levena and PBD2G was provided by Spirogen. A two-step process based on use of click chemistry reactive groups was used in which a lysine-based linker with a reactive group is first bound to the acceptor glutamines of the antibodies, followed by reaction with a second compound that includes the PBD1G (purchased from Levena) or PBD2G (given from Spirogen) and a complementary reactive group. To obtain the intermediate antibody bound to a reactive linker, 5 mg/mL mutant mAb was incubated with 20 equivalents of a reactive lysine-based linker (NH2-PEG-N3) (Click chemistry tools, cat. n. AZ101) per site of coupling and 2 U/mL BTG (Zedira, cat. n. T153) overnight at 37°C in PBS. The mAb-reactive linker conjugate was purified by affinity chromatography on protA. In order to produce the final ADC comprising a pyrrolobenzodiazepine (PBD) dimer, the azide-functionalized antibody above (2 mg/mL in PBS/1,2-propane-diol 50/50 v/v) was then incubated with 1.75 molar equivalent of DBCO-val-ala-PBD (DBCO-val-ala-PBD1G, purchased from Levena) or (DBCO-val-ala-PBD2G, given form SPIROGEN) or per site of coupling (the DBCO reacts with the azide). The mixture was incubated for 48-72h at RT with gentle agitation. Completion of the reaction was controlled by LC-ESI-MS (DAR >1.9). Excess of derivatized-PBD was removed by dialysis (MWCO=10 kDa), followed by purification by size exclusion chromatography (Superdex 200 10/300GL column, GEHealthcare). The final compounds were concentrated on Amicon 30K devices.

### LC/MS analysis of ADCs

Entire antibody was analyzed for DAR determination as previously described^11^. ADC products were eluted on a PLRP-S polymeric reverse-phase column (2.1 × 50 mm, 5 μm, 4000 Å, Agilent) heated at 80°C, at a flow rate of 0.35 mL/min, using the following gradient: 0–1 min, 5% B; 1–4 min, 5–50% B; 4–5 min, 50% B; 5–6 min, 50–5% B; 6–7 min, 5% B (A, water + 0.1% formic acid; B, acetonitrile + 0.1% formic acid). Analytes were ionized by electrospray and detected by a micro-TOF QII mass spectrometer (Bruker) operating in positive TOF-MS mode. Raw data were analyzed with Data Analysis software (Bruker), and deconvolution was performed using MaxEnt1, which could be replaced with OA-Analysis Engine^50^.

#### In vivo experiments

Mice had specific and opportunist pathogen free (SOPF) health status. Changes to litter were made every week for animals housed in a group. Animals housed in pairs were changed once every two weeks. The litter was composed of dusted poplar wood and the enrichment included a poplar brick as well as short cotton fibers. Mice were housed in ventilated cages with a maximum of five per cage of 500 cm^2^ on the ground. Animals were observed daily including weekends and holidays. Mice were housed in standardized temperature conditions (22°C ± 2°C) with relative humidity between 40 -75 % and had a 12-hour day and night daily lighting cycle. Both supply and exhaust air were HEPA-filtered and renewed 15-20 times per hour. Complete irradiated maintenance diet for rats, mice and hamsters and autoclaved tap water were available *ad libitum* in each cage. The acclimatization period before handling was a minimum of five days and the animals were handled only in dedicated rooms.

For the *in vivo* experiments in PDXs models performed at Xentech, the animal care and housing were in accordance with French regulatory legislation concerning the protection of animals used for scientific purposes. Mice were housed in groups of a maximum of seven animals during the acclimation period and a maximum of six animals during the experimental phase. Mice were housed inside individually ventilated cages (IVC) of polysulfone (PSU) plastic (mm 213 W x 362 D x 185 H, Allentown, USA) with sterilized and dust-free bedding cobs. Food and water were sterilized. Animals were housed under a light-dark cycle (14-hour circadian cycle of artificial light) and controlled room temperature and humidity. At request, the environmental conditions were monitored and the data were retained in the Central Animal House Archive. Drinking water was provided *ad libitum*. Each mouse was offered daily a complete pellet diet (150-SP-25, SAFE) throughout the study. The analytical certificate of animal food and water was retained at the CERFE premises. HBCx-5 and HBCx-34 breast tumor-bearing mice received estrogen diluted in drinking water. β-estradiol powder was suspended in 10% absolute ethanol and 90% distilled water to obtain the final concentration of 850 mg/l. This stock solution was added at 1/100 into the drinking water to obtain a final concentration of 8.5 mg/l. All animals were weighed before each experiment and identified by a unique pattern for ear punch numbering system. Each cage was identified by a paper tag indicating: cage number, mice strain and number, tumor code, date of experiment.

### *In vivo* MICAB1 efficacy in mouse tumor models

#### Disseminated Raji MICA*001 model

15×10^6^ Raji MICA*001 cells were injected intravenously (i.v.) into the tail vein in CB17 SCID females NOD (non-obese diabetic) SCID mice of 8-12 weeks old. The day of cell engraftment, different groups (n = 8–18 pool of two independent experiments) were treated with MICAB1-Fc-engineered Ab or IC. Mice were observed and weighed two to three times per week. All mice were used for this experiment without any exclusion. Mice were sacrificed by cervical dislocation when the weight loss was more than 20% or 15% (the day before weekend). End of protocol was at day 100. No adverse events were described. Group of animals for experiment 1: MICAB1 at 0.05 – 0.5 - 5 mg/kg (n=8) and IC at 5 mg/kg (n=8). Group of animals for experiment 2: MICAB1 at 0.5 – 2.5 mg/kg (n=10) and IC at 2.5 mg/kg (n=9).

#### Disseminated Raji MICA*001 and Raji MICA*008 i.p. models

15×10^6^ Raji MICA*001 cells with low level of MICA expression or Raji MICA*008 with medium level of MICA expression were injected intraperitoneally (i.p.) into NOD-SCID immunodeficient mice, which were then treated with single injection of i.v. 0.5 mg/kg MICAB1-Fc-engineered mAb or BAMO3 mAb or isotype control mAb (n= seven or eight mice per group). 24h after treatment, peritoneal cavity lavage (PCL) were performed to analyze cell number using microscope. The monitoring of mice weight was not performed. Six groups of animals: MICAB1 (one group for each tumor cell line), BAM03 (one group for each tumor cell line), IC (one group for each tumor cell line) and eight mice per group were used. A total of 44 8-9 week-old NOD SCID female mice were used. All mice were used for this experiment without any exclusion. Stratified randomization according to the mice age was used. End of protocol was at day 1. No adverse events were described.

#### Solid B16-F10 MICA*001 tumor model

3x10^5^ B16F10-MICA*001 tumor cells mixed with Matrigel (Corning) (1: 1) were inoculated s.c. in MICAgen mice at day 0. A total of 32 8–10-week-old MICAgen male or female mice were used. Stratified randomization was used when average tumor volume (TV) is ~61mm^3^ at day 5. The day of randomization 20 mice with 25mm^3^<TV <116mm^3^ were selected, 12 mice were excluded (four mice with TV<25mm^3^, three mice TV>116mm^3^, and five mice with malformed tumors). Two groups of mice (10 mice per group) were i.v. injected with 10 mg/kg of MICAB1-mIgG1a or IC-mIgG2a once a week for three weeks. Mice were monitored once to twice a week, the measured parameters including, TV and general health conditions. Tumor size was monitored with a digital caliper (Mitutoyo) and TVs were calculated as follows: TV = (length×width^2^) × (3.14/6). Body weight was not monitored for this experiment. Mice were sacrificed by cervical dislocation when tumors reach 1800 mm^3^ or when necrosis of tumors. End of protocol was at day 28 and end of experiment at day 35. No adverse events were observed.

### *In vivo* MICAB1 ADC efficacy in mouse tumor models

#### ADC-PBD1G efficacy

##### Xenogeneic model (HCT116)

A total of 32 10-week-old NUDE NMRI female mice were used. 2×10^6^ HCT-116 were mixed with Matrigel (1:1) (BD Biosciences) and s.c. inoculated in the right flank of mice. After nine days, stratified randomization was used when average TV is ~106 mm^3^. 12 mice were excluded (n = 1 TV<81mm^3^, n = 10 TV>136mm^3^, n = 1 malformed tumors) and 20 mice were selected (81mm^3^<TV<136mm^3^). Mice were randomized to obtain two homogeneous groups (n=10 mice per group) with a similar distribution of tumor sizes. Mice were treated with i.p. injection of MICAB1-PBD1G or IC-PBD1G at 0.05 mg/kg, once a week for three weeks. Injections were performed on anesthetized mice that were previously maintained for few minutes in a chamber containing 3.5% isoflurane. Mice were monitored once to two times a week, the measured parameters including TV and general health conditions. Tumor size was monitored with a digital caliper (Mitutoyo) and TV were calculated as above. No adverse events were described. Mice were sacrificed by cervical dislocation when tumors reach 2000 mm^3^ or when necrosis of tumors was observed. Weight monitoring was not performed for this experiment. End of protocol was at day 44.

##### PDX model (HBCx-5)

The anti-tumor activity of MICAB1-va-PBD1G was determined in (HBCx-5). A total of 46 female SHO mice (Crl:SHO-Prkdcscid Hrhr) aged 6–9 weeks old were used. The protocol has been previously described^38^. Briefly, 20 mm^3^ of tumor fragment from (tumors from donor mice, passage), was placed in the interscapular region, SC. Breast tumor-bearing mice received estrogen diluted in drinking water (β-estradiol, 8.5 mg/L), from the date of tumor implant to the date of inclusion (i.e. D0). Stratified randomization was used when average TV was ~130 mm^3^ at day 40. 20 mice were allocated, according to their TV, to give homogenous mean and median TV (60 mm^3^<TV<200 mm^3^) in each treatment arm and 26 mice were excluded with TV less than 60 mm^3^ or more than 200 mm^3^. Inclusion rate was 43%. Treatments were initiated 40 days post implantation of the tumor. A total of two groups were (10 mice per group) treated with IC-PBD1G (0.05 mg/kg qwk x 8 i.p.) or MICAB1-PBD1G (0.05 mg/kg qwk x 8 i.p.). All treatment doses were adjusted for body weight at time of dosing. In each experimental group, the mentioned dose was applied for all mice. Injected volume was around 100 μL. MICAB1-PBD1G or IC-PBD1G were i.p. administered on the enrolment day (i.e. D0). TV was evaluated by measuring perpendicular tumor diameters, with a caliper, twice a week during the whole experimental period. All animals were weighed at the same time as tumor size measurement. Relative body weight (RBW) loss was considered as an adverse effect of the treatment. Mice were observed every day for physical appearance, behavior and clinical changes. Mice were sacrificed by cervical dislocation when tumors reached 2000 mm^3^. Mice were sacrificed when necrosis of tumors was observed. No weight loss was reported. No adverse events were observed. The end of protocol was at 84 days.

##### Syngeneic models (B16F10 MICA*001)

A total of 90 eight-week-old C57BL6NRj female mice were used. 5×10^6^ B16F10 MICA*001 was mixed with Matrigel (1:1) (BD Biosciences) and s.c. inoculated in the right flank of mice. Stratified randomization was used when average TV was ~150 mm^3^ at day 4. The day of randomization, 70 mice were selected to obtain seven homogeneous groups with similar tumor size distribution (80 mm^3^<TV<188 mm^3^) (10 mice per group) and 20 mice were excluded (four mice with TV<80 mm^3^ and 16 mice with TV>188mm^3^). Mice were treated at day 4 with MICAB1-PBD1G or IC-PBD1G at doses of 0.1, 0.25, 0.5 mg/kg. 13 mice with complete tumor regression (from MICAB1-PBD1G treated groups) in addition to 10 new (naïve) C57Bl/6 females mice were used in three groups. Sample size was decided according to number of available mice in complete remission. Two groups with nine mice in complete remission and 10 naïve mice were re-challenged with s.c injection of 5×10^6^ B16F10MICA*001 tumor cells. Four mice in complete remission were not re-challenged and were used as tumor-free mice controls. Mice were monitored two to three times a week, the measured parameters including mouse weight, TV and general health conditions. Tumor size was monitored with a digital caliper (Mitutoyo) and TV were calculated as above. All mice were fine, without weight loss. Mice were euthanized by cervical dislocation when TV reached 2000 mm^3^ or tumor necrosis was observed. No adverse events were observed. The end of protocol was at 82 days.

#### ADC-PBD2G efficacy

##### Xenogeneic model (H1703)

A total of 33 eight-week-old NUDE NMRI female mice, were used. 7×10^6^ H1703 was mixed with Matrigel (1:1) (BD Biosciences) and s.c. inoculated in the right flank of mice. After nine days, stratified randomization was used when average TV was ~91 mm^3^.

The day of randomization, 27 mice was selected (55mm^3^<TV<141mm^3^) and six mice were excluded (four mice with TV<55mm^3^ and two mice with TV>141mm^3^). Mice were randomized to obtain three homogeneous groups (n=9 mice per group) with a similar distribution of tumor sizes. Mice were treated with single i.v. injection of MICAB2-PBD1G or IC-PBD2G at 0.3 mg/kg or vehicle. Injections were performed on anesthetized mice that were previously maintained for few minutes in a chamber containing 3.5% isoflurane. Mice were monitored once to two times a week, the measured parameters including TV, body weight and general health condition. Tumor size was monitored with a digital caliper (Mitutoyo) and TVs were calculated as above. No adverse events were described. Mice were sacrificed by cervical dislocation when tumors reached 1500 mm^3^–3000 mm^3^ or when necrosis of tumors was observed. No body weight loss was described for this experiment. The end of protocol was at day 131.

##### Xenogeneic model (HCT116)

A total of 70 eight-week-old NUDE NMRI female mice were used. 2×10^6^ HCT116 was mixed with Matrigel (1:1) (BD Biosciences) and s.c. inoculated in the right flank of mice. After five days, stratified randomization was used when average TV was ~106 mm^3^. The day of randomization, 63 mice were selected (55 mm^3^<TV<171 mm^3^). Seven mice were excluded (TV<55mm^3^). Mice were randomized to obtain seven homogeneous groups (n=9 mice per group, seven groups) with a similar distribution of tumor sizes. Mice were treated with single i.v. injection of MICAB2-PBD1G (at three indicated dose) or IC-PBD2G (at three indicated dose) or vehicle. Doses were 0.5 or 1 or 2 mg/kg. Injections were performed on anesthetized mice that were previously maintained for few minutes in a chamber containing 3.5% isoflurane. Mice were monitored once to two times a week, the measured parameters including, TV, body weight and general health conditions. Tumor size was monitored with a digital caliper (Mitutoyo) and TVs were calculated as above. No adverse events were described excepted for one mouse from the MICAB1-PBD2G group. This mouse was sacrificed at day 47 because of oedema. Mice were sacrificed by cervical dislocation when tumors reached 1500 mm^3^-2000 mm^3^ or when necrosis of tumors was observed. No body weight loss was described for this experiment. The end of protocol was at day 83.

##### PDXs models (HBCx-5 and HBCx-34)

The anti-tumor activity of MICAB1-va-PBD2G was determined in the Her2+ breast cancer PDX model (HBCx-5) and ER+PR+HER2-breast cancer PDX model (HBCx-34).

#### HBCx-5 model

A total of 49 female nude mice aged 6-9 weeks old were used. The protocol has been previously described^38^. Briefly, 20 mm^3^ of tumor fragment from (tumors from donor mice, passage (n-1)) was placed in the interscapular region, SC. Breast tumor-bearing mice received estrogen diluted in drinking water (β-estradiol, 8.5mg/L), from the date of tumor implant to the end of the experiment. Stratified randomization was used when average TV was ~140 mm^3^ at day 40. 20 mice were allocated according to their TV to give homogenous mean and median TV (75 mm^3^<TV<220 mm^3^) in each treatment arm and 29 mice were excluded with TV less than 75 mm^3^ or more than 220 mm^3^. Inclusion rate was 41%. Treatments were initiated 40 days post implantation of the tumor. Two groups (10 mice per group) were treated with a single i.v. injection of MICAB1-PBD2G (0.5 mg/kg) or vehicle. All treatment doses were adjusted for body weight at time of dosing. In each experimental group, the mentioned dose was applied for all mice. Injected volume was around 100 μL. MICAB1-PBD2G or vehicle were i.v. administered on the enrolment day (i.e. D0). TV was evaluated by measuring perpendicular tumor diameters, with a caliper, twice a week during the whole experimental period. All animals were weighed at the same time as tumor size measurement. RBW loss was considered as an adverse effect of the treatment. Mice were observed every day for physical appearance, behavior and clinical changes. Mice were sacrificed by cervical dislocation when tumors reached 2000 mm^3^ or when necrosis of tumors was observed. No weight loss was reported. No adverse events were observed. The end of protocol was at 79 days.

#### HBCx-34 model

A total of 47 female nude mice (Hsd:Athymic Nude-Foxn1nu) aged 6-9 weeks old were used. The protocol has been previously described^38^. Briefly, 20 mm^3^ of tumor fragment from (tumors from donor mice, passage (n-1)) was placed in the interscapular region, SC. Breast tumor-bearing mice received estrogen diluted in drinking water (β-estradiol, 8.5mg/L), from the date of tumor implant to the date of inclusion (i.e. D0). Stratified randomization was used when average TV was ~140 mm^3^ at day 38. 19 mice were allocated according to their TV to give homogenous mean and median TV (108 mm^3^<TV<288 mm^3^) in each treatment arm. 28 mice were excluded with TV less than 108 mm^3^ or more than 288 mm^3^. Inclusion rate was 41%. Treatments were initiated 38 days post implantation of the tumor. Mice were treated with i.v. single injection of MICAB1-PBD2G (0.5 mg/kg, 10 mice per group) or vehicle (nine mice per group). All treatment doses were adjusted for body weight at time of dosing. In each experimental group, the mentioned dose was applied for all mice. Injected volume was around 100 μL. MICAB1-PBD2G or vehicle were i.v. administered on the enrolment day (i.e. D0). TV was evaluated by measuring perpendicular tumor diameters, with a caliper, twice a week during the whole experimental period. All animals were weighed at the same time as tumor size measurement. RBW loss was considered an adverse effect of the treatment. Mice were observed every day for physical appearance, behavior and clinical changes. Mice were sacrificed by cervical dislocation when tumors reached 1000 mm^3^ or when necrosis of tumors was observed. No weight loss was reported. No adverse events were observed. The end of protocol was at day 81.

##### MCA induced tumor models

#### Efficacy of MICAB1-PBD2G on 3-MCA model

A total of 90 7-14-week-old, transgenic male mice expressing human MICA*007 allele (MICAgen mice)^34^ were induced with 100 μg 3-MCA injected s.c. Mice were randomized into five groups (*n*=15 per group) when tumors reached ~150 mm^3^. 75 mice were selected (105 mm^3^<TV<288 mm^3^) and 15 mice were excluded with TV less than 105 mm^3^. Stratified randomization was used when average TV was ~150 mm^3^ to obtain homogeneous groups with similar tumor size distribution. Around 80-100 days after MCA treatment, mice were injected i.v. with MICAB1-PBD2G or IC-PBD2G at 0.5 mg/kg or 2 mg/kg or the vehicle (30 mM histidine, 200 mM sorbitol and 0.02% Tween 20 in PBS1X, pH=6) once on the day of randomization. Treatments were not blinded. Mice were observed and TV were evaluated by measuring perpendicular tumor diameters, with a caliper, one to twice a week during the whole experimental period. Body weight was not monitored for this experiment. Mice were sacrificed by cervical dislocation when tumors reached 1400-2100 mm^3^ or when necrosis of tumors was observed. No adverse effects were observed. The end of the protocol was at day 70 after first treatment.

##### Comparison between MICAB1-PBD2G and MICAB1-mIgG2a

The experimental design was the same as previously described. A total of 76 mice were used. Stratified randomization was used when average TV reached 120 mm^3^ to obtain homogeneous TV distribution. 52 mice were selected (120 mm^3^<TV<300 mm^3^) and 24 mice without tumor growth were excluded. Mice were randomized into four groups (n=13 per group), and injected i.v. once with MICAB1-PBD2G or IC-PBD2G at 2 mg/kg at 10mg/kg or three times with MICAB1-mIgG2a or IC-mIgG2a at 10 mg/kg per week. The first injection was on the day of randomization. Treatments were not blinded. Mice were observed and TV was evaluated by measuring perpendicular tumor diameters with a caliper and body weight was monitored once to twice a week during the whole experimental period. No weight loss was observed. No adverse effects were observed. Mice were sacrificed by cervical dislocation when tumors reached 1400 mm^3^ or when necrosis of tumors was observed. The end of protocol was at day 60 after first treatment.

#### Quantification and statistical analysis

Details on statistics used can be found in figure legends. All statistical analyses were performed using R version 4.0.3. The effects of the antibody and its concentration on the percentage of lysis, the MedFI and the phagocytosis are evaluated with a two-ways Anova : x~Antibody + Concentration+Antibody: Concentration

Data are shown with means and error bars showing the SD or median and range. Data from one representative experiment tested in simplicate are shown without SD or range.

Tumor growth models are analyzed with a linear mixed-effects model (lmer):

Volume ~Antibody + Time + Antibody:Time + (1|Mouse.ID). Antibody, time and the interaction are fixed effects. Mouse. ID is the random effect. ANOVA was performed on this model to evaluate the significance of each fixed effect.

Pairwise differences of least squares mean for the factor antibody were computed. Confidence intervals and p-values were based on the t-distribution using degrees of freedom based on Kenward-Roger methods. The difference of percent of CD137+ NK cells between MICAB Fc-engineered and Isotype or Fc silent was evaluated by the Friedman rank sum test. The difference of cell counts between antibodies in the two Raji models (Raji *01, Raji*08) was evaluated by the Kruskal-Wallis rank sum test and pairwise comparisons are estimated by Wilcoxon rank sum exact tests. Kaplan Meier curves were used for survival representations and log-rank tests were applied to evaluate the difference of survival. Significance was assumed with *p < 0.05; **p ≤ 0.01; ***p < 0.001, ****p < 0.0001.

## Abreviations

2A9-MICA: Bispecific antibody consists of human MICA extracellular region and a single-chain antibody fragment (scFv) that targets BCMA
Ab: Antibody
ADC: Antibody-drug conjugates
ADCC: Antibody dependent cellular cytotoxicity
ADCP: Antibody-dependent cellular phagocytosis
B2-OKT3: Bispecific T-cell engagers created in the tandem single-chain variable fragment BiTE format that targets MICA on tumor cells and CD3ε on human T cells
BCMA: B-cell maturation antigen
BiTE: Bispecific T cell engager
BTG: Bacterial transglutaminase
CAR-NK: Chimeric antigen receptor on natural killer cells
E2F: E2 Factor protein
EGF-R: Epidermal growth factor receptor
ER: Estrogen receptor
H&N: Head and neck
HBCx-34: Human breast cancer xenografts number 34
HBCx-5: Human breast cancer xenografts number 5
HER2: Human epidermal growth factor 2
HNSCC: Head Neck squamous cell carcinoma
IC: Isotype control
IHC: Immunohistochemical staining
JZC01: Bispecific antibody consisting of MICA and an anti- VEGFR2 single chain antibody fragment
mAb: Monoclonal antibody
MCA: Methylcholanthrene
mg/kg: Milligram per kilogram of mice
MHC: Major histocompatibility complex
MICA*001 or MICB*002: MHC class I chain-related protein A allele 001 or B allele 002
MICA and MICB or MICA/B: MHC class I chain-related protein A and B
MICAgen: MICA transgenic mouse
NOD-SCID: Non Obese Diabetic Severe Combined ImmunoDeficiency
OKT3: CD3ε binding single-chain variable fragment (scFv)
p53: Tumor protein 53
PBD: Pyrrolobenzodiazepine
PBD1G, SGD-1882: First-generation PBD
PBD2G, SG-3249: Second-generation PBD
PBMC: Peripheral blood mononuclear cell
PDX: Patient-derived xenografts
TNBC: Triple negative breast cancer
Trop-2: Tumor-associated calcium signal transducer 2
VEGFR2: Vascular endothelial growth factor receptor 2

## Figures and Tables

**Table 1:** Generation of a wide repertoire of anti-MICA and MICB monoclonal antibodies (mAbs) from two independent immunizations.

Immunization	Immunogen	Selected mAb
1.	MICA*019-Fc recombinant protein	MICAB1 MICAB2 MICA1 MICA2
2.	Mix of C1R-MICA*001. MICA*004. MICA*007. MICA*008 transfected cells	MICAB4 MICAB5 MICAB6 MICA3 MICA4 MICA5 MICA6 MICA7

MICA= antibody binding to MICA only. MICAB= antibody binding to both MICA and MICB.

**Table 2:** Immunohistochemical analyses of patient-derived xenografts (PDX) tissues for MICA/B expression.

Cancer subtype	N (PDXs)	N (QS≥ 100)	N (QS≥ 200)
Breast no TNBC	13	7	4
Breast TNBC	25	9	7
Lung (NSCLC)	24	11	2
Colon	21	3	1
Liver	20	4	1
Kidney	7	3	3
Melanoma	6	4	1
Endometrium	4	2	2
Lung (SCLC)	7	0	0
Brain	6	1	0
Prostate	6	1	0
Ovarian	3	2	0
Pancreatic	3	0	0
Liposarcome	1	0	1

N: number of PDX samples. QS: quick score = staining intensity x % of stained cells. Staining intensity from 0 to 3. Stained cells from 0 to 100.

**Table 3:** MICA and MICB protein expression in formalin-fixed, paraffin-embedded (FFPE) tissues from MICAgen mouse as determined by immunohistochemistry. This table summarizes MICA/MICB expression observed on epithelial cells in most of the MICAgen mice tissues using Mia4 mAb.

	IHC analysis of MICA expression on MICAGEN mice
Lymph node	Membranous staining on less than 1% of cells and background noise.
Stomach	Membranous and cytoplasmic staining in the mucosa.
Colon	Weak membranous and cytoplasmic staining in the mucosa.
Prostate	Membranous and cytoplasmic staining in the epithelium of the prostate glands. Membranous staining in 2% of the epithelial cells of the prostatic urethra.
Lung	Membranous and cytoplasmic staining in the epithelium of the bronchiioles and on 5% of pneumocytes.
Testis	Strong membranous and cytoplasmic staining in the spermatogonia in all the seminiferous ducts.
Kidney	Membranous staining on tubules. No staining on glomerulus.
Ovary	Membranous and cytoplasmic staining on oocytes and in the stroma.
Fallopian tubes	Membranous and cytoplasmic staining on the epithelium of the fallopian tubes.
Uterus	Membranous (and slightly cytoplasmic) staining on endometrial glands (epithelial cells). Staining also observed in the vascular endothelium.
Liver	Membranous staining on hepatocytes and on the epithelium of some central veins.

## Data Availability

Figshare: Targeting MICA/B with cytotoxic therapeutic antibodies leads to tumor control. http://dx.doi.org/10.6084/m9.figshare.c.5549109.v1^51^. This project contains the following underlying data: - Fig. 1a (NDPI files containing MICA/MICB specific positive staining with Mia4 Ab compared to IC in two breast cancer slides) - Fig. 1b (NDPI files containing MICA/MICB staining on tissues slides from healthy donors) - Fig. 1c (NDPI and mrxs files containing MICA/MICB staining on tumor slides from head and neck squamous cell carcinoma (HNSCC), mesothelioma, ovarian cancer, endometrial cancer, breast cancer and melanoma) - Fig. 1d (XLSX file containing number of patients in different indication scored based on proportion of MICA/B positive cells) - Fig. 1e (XLSX file containing number of patients with different subtypes of breast cancer scored based on proportion of MICA/MICB positive cells) - Fig. 1f (MRXS files containing membranous MICA/MICB positive staining on two breast cancer slides at different scores) - Fig. 2a (XLSX file containing BCM (background corrected mean fluorescence intensity) of MICAB1 at 10 μg/ml with different allele of MICA/B) - Fig. 2b (XLSX file containing % specific lysis on C1R MICA*001 and C1R MICB*002 at indicated concentration of MICAB1 Fc-engineered vs IC) - Fig. 2C (XLSX file containing % CD137 among total of NK cells at indicated conditions and FCS files containing data of CD137 expression in NK as measured by quantitative flow cytometry) - Fig. 2d-Lysis (XLSX file containing data of % specific lysis on A549 and Raji MICA*001 at indicated conditions) - Fig. 2d-MFI (XLSX file containing data of MFI at indicated conditions and - FCS files containing data of mAb binding (MICAB1 Ab or cetuximab or rituximab or IC) at the cell surface of A549 or Raji MICA *001 as measured by quantitative flow cytometry) - Fig. 2e (XLSX file containing data of macrophage intracellular fluorescence intensity as measured by EnSpire at indicated conditions) - Fig. 3a (XLSX files containing data of mice survival in different groups and data of body weight monitoring) - Fig. 3b (XLSX file containing data of MFI at indicated conditions and FCS files of tumor cell counts in peritoneal cavity lavage of mice injected with Raji MICA*01 or MICA*08 and treated with MICAB1 or BAMO3 or IC as measured by quantitative flow cytometry) - Fig. 3c (XLSX file containing data of tumor volume monitoring in indicated groups) - Fig. 3d (XLSX file containing data of doubling time (day) for tumors in mice treated with IC or MICAB1) - Fig. 4a (XLSX files containing data of MFI in Raji wt at indicated conditions and data of MFI in Raji MICA*001 at indicated conditions and FCS files of dye fluorescence upon anti-MICA mAb or IC internalization in Raji wt or Raji MICA*001 as measured by quantitative flow cytometry) - Fig. 4c (XLSX file containing data of tumor (HCT116 xenograft model) volume monitoring in mice treated with IC-PBD1G or MICAB1-PBD1G) - Fig. 4d (XLSX files containing data of tumor (HBCx-5 PDX model) volume monitoring in mice treated with IC-PBD1G or MICAB1-PBD1G and data of body weight monitoring in mice treated with IC-PBD1G or MICAB1-PBD1G) - Fig. 4e (XLSX files containing data of tumor (B16F10 MICA*001) volume monitoring in mice treated with vehicle or IC-PBD1G or MICAB1-PBD1G, data of body weight monitoring in mice treated with IC-PBD1G or MICAB1-PBD1G, data of mice survival in different groups and tumor volume monitoring in cured or naïve mice challenged with B16F10 MICA*001) - Fig. 5a (XLSX files containing data of tumor (H1703 xenograft model) volume monitoring in mice treated with IC-PBD2G or MICAB1-PBD2G or vehicle and data of body weight monitoring) - Fig. 5b (XLSX files containing data of tumor (HC116 xenograft model) volume monitoring in mice treated with IC-PBD2G or MICAB1-PBD2G or vehicle and data of body weight monitoring) - Fig. 5c (XLSX files containing data of tumor (HBCx-5 PDX model) volume monitoring in mice treated with vehicle or MICAB1-PBD2G, data of tumor (HBCx-34 PDX model) volume monitoring in mice treated with vehicle or MICAB1-PBD2G and data of body weight monitoring in these mice) - Fig. 5d (NDPI files containing MICA expression on FFPE tissues slides from testis, liver and lung of MICAgen mice) - Fig. 5e (XLSX file containing data of MFI with MICAB1-APC or IC-APC and FCS files of MICA expression in MCA-induced tumors after MICAB1-APC or IC-APC staining as measured by quantitative flow cytometry) - Fig. 5f (XLSX file containing data of tumor (MCA-induced tumors) volume monitoring in mice treated with vehicle or IC-PBD2G or MICAB1-PBD2G) - Fig. 5g (XLSX files containing data of tumor (MCA-induced tumors) volume monitoring in mice treated with vehicle or MICAB1-PBD2G or mIgG2a-IC or mIgG2a-MICAB1 and data of body weight monitoring in these mice) This project contains the following underlying data: - XLSX file containing number of patients with different subtypes of breast cancer scored based on proportion of MICA/MICB positive cells Data are available under the terms of the Creative Commons Attribution 4.0 International license (CC-BY 4.0).

## Amendments from Version 1

In this revised version of the manuscript, we give more information in the introduction part. We thus had to update the reference section. We also have modified the color code of the Figure 4e and added in the methods a table for cell lines and a list for abbreviations.

**Any further responses from the reviewers can be found at the end of the article**
