# Supplementary material for: Targeting MICA/B with cytotoxic therapeutic antibodies leads to tumor control
Source: Open Res Eur. 2021 Oct 27;1:107. Originally published 2021 Sep 13. [Version 2] doi: 10.12688/openreseurope.13314.2 (PMC7613279; doi:10.12688/openreseurope.13314.2)
Supplement: Peer review history

# Peer review: Reviewer response for version 2

**DOI:** 10.21956/openreseurope.15342.r27925

**Published:** 2021-11-15

**Authors:** Jacques Zimmer

**Affiliations:** 1 Department of Infection and Immunity, Luxembourg Institute of Health (LIH), Esch-sur-Alzette, Luxembourg

The authors satisfactorily addressed all concerns, and so I have no further comments to make.

Is the study design appropriate and does the work have academic merit?

Yes

Is the work clearly and accurately presented and does it cite the current literature?

Yes

If applicable, is the statistical analysis and its interpretation appropriate?

Yes

Are all the source data underlying the results available to ensure full reproducibility?

Yes

Are the conclusions drawn adequately supported by the results?

Yes

Are sufficient details of methods and analysis provided to allow replication by others?

Yes

Reviewer Expertise:

natural killer cells; major histocompatibility complex

I confirm that I have read this submission and believe that I have an appropriate level of expertise to confirm that it is of an acceptable scientific standard.

# Peer review: Reviewer response for version 1

**DOI:** 10.21956/openreseurope.14384.r27610

**Published:** 2021-10-04

**Authors:** Roberta Castriconi

**Affiliations:** 1 Department of Experimental Medicine (DIMES), University of Genova, Genoa, Italy

The authors have addressed a crucial and challenging issue in the context of mAb-mediated therapies against tumors, and represented by a general low efficacy of these approaches in solid tumors. The study proposes a novel therapeutic strategy based on the targeting of MICA and MICB molecules with mAb conjugated with a second generation of pyrrolobenzodiazepine less toxic for the target cells (MICAB1-PBD2G). The study, based on an array of *in vivo* models of solid tumors, including MCA-induced fibrosarcoma in an immunocompetent MICA-transgenic mouse model, and PDX, shows that MICAB1-PBD2G is a very effective approach. All the experiments have been well conducted and well described with an appropriate statistical analysis. All the sections of the manuscript are well written and complete. Overall, the study represents an important step forward in optimizing the therapies of solid tumors.

Is the study design appropriate and does the work have academic merit?

Yes

Is the work clearly and accurately presented and does it cite the current literature?

Yes

If applicable, is the statistical analysis and its interpretation appropriate?

Yes

Are all the source data underlying the results available to ensure full reproducibility?

Yes

Are the conclusions drawn adequately supported by the results?

Yes

Are sufficient details of methods and analysis provided to allow replication by others?

Yes

Reviewer Expertise:

NK cells, neuroblastoma, immunotherapies

I confirm that I have read this submission and believe that I have an appropriate level of expertise to confirm that it is of an acceptable scientific standard.

# Peer review: Reviewer response for version 1

**DOI:** 10.21956/openreseurope.14384.r27601

**Published:** 2021-09-27

**Authors:** Jacques Zimmer

**Affiliations:** 1 Department of Infection and Immunity, Luxembourg Institute of Health (LIH), Esch-sur-Alzette, Luxembourg

In this manuscript, Bléry *et al*. report on the generation and evaluation of MICA/B-targeting antibodies that are tested *in vitro* and *in vivo* in a large panel of cancer models. Overall, the efficiency of the antibody MICAB1 was not sufficient *in vivo* against a solid tumor (melanoma), and this was the reason why antibody-drug conjugates (ADC) were developed and used in,cancer models, among them patient-derived xenografts and carcinogen-induced tumors. The ADC lead to tumor control in the various configurations tested.

 The article is sound, well performed and timely, as the targeting of some of the ligands of the NK and T cell activating receptor NKG2D, namely MICA/MICB, have not yet been tested with the ADC method but could represent, as this work shows, an interesting addition to the immunotherapeutic arsenal against cancer, and particularly solid tumors.

 Although this manuscript is globally very relevant and interesting, some items could be clarified or a bit modified:

1. The Introduction is very short, especially in the last paragraph, where some background information about the toxin PBD, the associated technology and the mechanism of action could be added.
2. There are many abbreviations throughout the article that are not all explained at first appearance; adding a table with all of them listed and explained (for example, ADC: antibody-drug conjugate, etc) would help.
3. Likewise, a table of the cell lines used (there are a lot) with their name, the species of origin, where they come from and what is their histology (for example: melanoma) and another table of the mouse models used would help.
4. The Mia4 antibody clearly distinguishes normal from cancerous tissues with a bright positive staining of the latter. Would it be possible to demonstrate the specificity of this antibody for MICA/MICB by including a reference antibody for comparison, such as the clone 6D4 or another one (for the IHC and/or for flow cytometry by staining MICA/MICB-transfected C1R cells with both antibodies)?
5. What was the rationale for the choice of C1R cells for the transfection experiments? The absence of endogenous MICA/MICB, the low expression of HLA class I, other?
6. When the MICAB1-Fc engineered antibody was used, was the isotype control in all cases also Fc-engineered? This is stated in one figure legend but not everywhere in the text.
7. Could the authors indicate one or two reference(s) for the use of CD137 as a NK cell-activation marker?
8. In figure 2b, ADCC induced by MICAB1-Fc engineered was compared with the isotype control. Was the "basic" level of NK cell cytotoxic activity (natural killing) evaluated in parallel?
9. In figure 2c, in the C1R-neo condition, 25% of the cells are CD137+, although there is no ligand for the antibody on this target cell line (in principle).
10. The *in vitro* experiments were often done only twice.
11. Figure 4: the color code in the right part of figure 4e is not optimal.

Minor points

1. There are some typos throughout the manuscript or sentences whose structure could be slightly modified.
2. Adverse events in the mouse experiments: was there a precise list that was checked? Body weight surveillance is considered frequently as a standard in invasive mouse experiments.
3. What were the "general health conditions" controlled. Here, a table might be more precise.
4. On page 20, what is the vehicle for the control groups of the *in vivo* models? The same than in the MCA model?
5. In the legend to figure 2, is the value p=0.14 considered as significant, or is this a typo?

Is the study design appropriate and does the work have academic merit?

Yes

Is the work clearly and accurately presented and does it cite the current literature?

Yes

If applicable, is the statistical analysis and its interpretation appropriate?

Yes

Are all the source data underlying the results available to ensure full reproducibility?

Yes

Are the conclusions drawn adequately supported by the results?

Yes

Are sufficient details of methods and analysis provided to allow replication by others?

Yes

Reviewer Expertise:

natural killer cells; major histocompatibility complex

I confirm that I have read this submission and believe that I have an appropriate level of expertise to confirm that it is of an acceptable scientific standard.

# Response

**Published:** 2021-10-16

**Authors:** Emilie Narni-Mancinelli

**Affiliations:** INSERM, France

*Minor points*

1. *There are some typos throughout the manuscript or sentences whose structure could be slightly modified.*
2. *Adverse events in the mouse experiments: was there a precise list that was checked? Body weight surveillance is considered frequently as a standard in invasive mouse experiments.*
3. *What were the "general health conditions" controlled. Here, a table might be more precise.*
**Author Response: **Limitations related to weight loss, appearance and behavior of the animals (prostration, prickly hair, isolation, breathing difficulties (dyspnea), paralysis, etc.) were the criteria used to monitor the general health conditions.
4. *On page 20, what is the vehicle for the control groups of the in vivo models? The same than in the MCA model?*
**Author Response:** Yes the vehicle was composed of 30 mM histidine, 200 mM sorbitol and 0.02% Tween 20 in PBS1X, pH=6.
5. *In the legend to figure 2, is the value p=0.14 considered as significant, or is this a typo?*
**Author Response:** No p=0.14 is not significant as mentioned in the legend « Pairwise comparisons conclude to a significant difference between MICAB1 Fc-engineered and the isotype control (p value=1.3e-05 in A549 and 1.7e-09 in Raji-MICA*001) or Rituximab (p-value = 0.03) but not with Cetuximab (p-value=0.14))  

*Major points:  *

1. *The Introduction is very short, especially in the last paragraph, where some background information about the toxin PBD, the associated technology and the mechanism of action could be added.*
**Author Response:** The introduction has been revised accordingly.
2. *There are many abbreviations throughout the article that are not all explained at first appearance; adding a table with all of them listed and explained (for example, ADC: antibody-drug conjugate, etc) would help.*
**Author Response:** A table has been added in the revised manuscript as follow:   2A9-MICA Bispecific antibody consists of human MICA extracellular region and a single-chain antibody fragment (scFv) that targets BCMA Ab Antibody ADC Antibody-drug conjugates ADCC Antibody dependent cellular cytotoxicity ADCP Antibody-dependent cellular phagocytosis B2-OKT3 Bispecific T-cell engagers created in the tandem single-chain variable fragment BiTE format that targets MICA on tumor cells and CD3ε on human T cells BCMA B-cell maturation antigen  BiTE Bispecific T cell engager BTG Bacterial transglutaminase CAR-NK Chimeric antigen receptor on natural killer cells E2F E2 Factor protein EGF-R Epidermal growth factor receptor ER Estrogen receptor H&N Head and neck HBCx-34 Human breast cancer xenografts number 34 HBCx-5 Human breast cancer xenografts number 5 HER2 Human epidermal growth factor 2 HNSCC Head Neck squamous cell carcinoma IC Isotype control IHC Immunohistochemical staining JZC01  Bispecific antibody consisting of MICA and an anti- VEGFR2 single chain antibody fragment mAb Monoclonal antibody MCA Methylcholanthrene mg /kg Milligram per kilogram of mice MHC Major histocompatibility complex MICA*001 or MICB*002 MHC class I chain-related protein A allele 001 or B allele 002 MICA and MICB or MICA/B MHC class I chain-related protein A and B MICAgen MICA transgenic mouse NOD-SCID Non Obese Diabetic Severe Combined ImmunoDeficiency OKT3 CD3ε binding single-chain variable fragment (scFv) p53 Tumor protein 53 PBD Pyrrolobenzodiazepine PBD1G, SGD-1882 First-generation PBD PBD2G, SG-3249 Second-generation PBD PBMC Peripheral blood mononuclear cell PDX Patient-derived xenografts TNBC Triple negative breast cancer Trop-2 Tumor-associated calcium signal transducer 2 VEGFR2 Vascular endothelial growth factor receptor 2
3. *Likewise, a table of the cell lines used (there are a lot) with their name, the species of origin, where they come from and what is their histology (for example: melanoma) and another table of the mouse models used would help.*
**Author Response: **A table has been added in the revised manuscript as follow:   Cell line Characteristics A549 Human lung cancer cells B16F10 Mouse melanoma  cells C1R Human B lymphoblast H1703 Human lung carcinoma cells HBCx-34 HER-2low luminal breast cancer patient-derived xenografts HBCx-5 HER-2high breast cancer patient-derived xenografts HCT-116 Human colon carcinoma cells MCA-induced tumors Methylcholanthrene induced tumors Raji Human B-cell lymphoma cells.
4. *The Mia4 antibody clearly distinguishes normal from cancerous tissues with a bright positive staining of the latter. Would it be possible to demonstrate the specificity of this antibody for MICA/MICB by including a reference antibody for comparison, such as the clone 6D4 or another one (for the IHC and/or for flow cytometry by staining MICA/MICB-transfected C1R cells with both antibodies)?*
**Author Response**: IHC was performed on RAJI (negative control) and C1R MICA*001 FFPE cell lines with the commercial clone BAMO1 or our proprietary clone (Mia4). Both clones strongly stain C1R MICA*001 cells and not Raji cells.. It is important to note that we were not able to detect endogenous MICA/B expression on cell lines or cancer tissues with the commercial clone BAMO1, highlighting the poor sensitivity of this antibody for IHC application. To our knowledge, there is no other commercially available anti-MICA/B for IHC application on FFPE samples.
5. *What was the rationale for the choice of C1R cells for the transfection experiments? The absence of endogenous MICA/MICB, the low expression of HLA class I, other?*
**Author Response:**C1R cells were selected based on both the absence of HLA class I and no detectable cell surface MICA/MICB . (STORKUS, W. et all PNAS 1989, Groh, V. et al Science 1998, Steinle, A. et al Immunogenetics 2001)
6. *When the MICAB1-Fc engineered antibody was used, was the isotype control in all cases also Fc-engineered? This is stated in one figure legend but not everywhere in the text.*
**Author Response: **We confirm that when the MICAB1-Fc engineered antibody was used, isotype control was also Fc engineered in all cases as indicated in the Methods of the revised manuscript.
7. *Could the authors indicate one or two reference(s) for the use of CD137 as a NK cell-activation marker?*
**Author Response: **Raghvendra M Cancer Therapy (2017) ( **DOI:** 10.1158/1078-0432.CCR-16-0879) Voabil et al. Nature Medicine 27, no 7 (2021): 1250 61 (DOI: org/10.1038/s41591-021-01398-3)
8. *In figure 2b, ADCC induced by MICAB1-Fc engineered was compared with the isotype control. Was the "basic" level of NK cell cytotoxic activity (natural killing) evaluated in parallel?*
**Author Response: **Basic level of NK cell cytotoxic activity is added in the figure below. This basal activity is similar to the one observed at the lowest mAb concentration.
